# Exome sequencing reveals *IFT172* variants in patients with non-syndromic cholestatic liver disease

**DOI:** 10.1371/journal.pone.0288907

**Published:** 2023-07-20

**Authors:** Magdaléna Neřoldová, Elżbieta Ciara, Janka Slatinská, Soňa Fraňková, Petra Lišková, Radana Kotalová, Janka Globinovská, Markéta Šafaříková, Lucie Pfeiferová, Hana Zůnová, Lenka Mrázová, Viktor Stránecký, Alena Vrbacká, Ondřej Fabián, Eva Sticová, Daniela Skanderová, Jan Šperl, Marta Kalousová, Tomáš Zima, Milan Macek, Joanna Pawlowska, A. S. Knisely, Stanislav Kmoch, Milan Jirsa

**Affiliations:** 1 Institute for Clinical and Experimental Medicine, Prague, Czech Republic; 2 Institute of Medical Biochemistry and Laboratory Diagnostics, First Faculty of Medicine, Charles University and General University Hospital in Prague, Prague, Czech Republic; 3 Department of Medical Genetics, The Children’s Memorial Health Institute, Warsaw, Poland; 4 Department of Ophthalmology, First Faculty of Medicine, Charles University and General University Hospital in Prague, Prague, Czech Republic; 5 Department of Pediatrics and Inherited Metabolic Diseases, First Faculty of Medicine, Charles University and General University Hospital in Prague, Prague, Czech Republic; 6 Department of Pediatrics, Second Faculty of Medicine, Charles University and Faculty Hospital Motol, Prague, Czech Republic; 7 Department of Pediatrics, Hospital Poprad, Poprad, Slovak Republic; 8 Department of Informatics and Chemistry, University of Chemistry and Technology in Prague, Prague, Czech Republic; 9 Department of Biology and Medical Genetics, Second Faculty of Medicine, Charles University and Motol University Hospital, Prague, Czech Republic; 10 Department of Pathology and Molecular Medicine, 3rd Faculty of Medicine, Charles University and Thomayer Hospital, Prague, Czech Republic; 11 Department of Pathology, Faculty of Medicine and Dentistry, Palacky University Olomouc and Faculty Hospital, Olomouc, Czech Republic; 12 Department of Gastroenterology, Hepatology, Nutritional Disorders and Pediatrics, The Children’s Memorial Health Institute, Warsaw, Poland; 13 Diagnostik- und Forschungsinstitut für Pathologie, Medizinische Universität Graz, Graz, Austria; Medizinische Fakultat der RWTH Aachen, GERMANY

## Abstract

**Background and aim:**

Gene defects contribute to the aetiology of intrahepatic cholestasis. We aimed to explore the outcome of whole-exome sequencing (WES) in a cohort of 51 patients with this diagnosis.

**Patients and methods:**

Both paediatric (n = 33) and adult (n = 18) patients with cholestatic liver disease of unknown aetiology were eligible. WES was used for reassessment of 34 patients (23 children) without diagnostic genotypes in *ABCB11*, *ATP8B1*, *ABCB4* or *JAG1* demonstrable by previous Sanger sequencing, and for primary assessment of additional 17 patients (10 children). Nasopharyngeal swab mRNA was analysed to address variant pathogenicity in two families.

**Results:**

WES revealed biallelic variation in 3 ciliopathy genes (*PKHD1*, *TMEM67* and *IFT172*) in 4 clinically unrelated index subjects (3 children and 1 adult), heterozygosity for a known variant in *PPOX* in one adult index subject, and homozygosity for an unreported splice-site variation in *F11R* in one child. Whereas phenotypes of the index patients with mutated *PKHD1*, *TMEM67*, and *PPOX* corresponded with those elsewhere reported, how *F11R* variation underlies liver disease remains unclear. Two unrelated patients harboured different novel biallelic variants in *IFT172*, a gene implicated in short-rib thoracic dysplasia 10 and Bardet-Biedl syndrome 20. One patient, a homozygote for *IFT172* rs780205001 c.167A>C p.(Lys56Thr) born to first cousins, had liver disease, interpreted on biopsy aged 4y as glycogen storage disease, followed by adult-onset nephronophthisis at 25y. The other, a compound heterozygote for novel frameshift variant *IFT172* NM_015662.3 c.2070del p.(Met690Ilefs*11) and 2 syntenic missense variants *IFT172* rs776310391 c.157T>A p.(Phe53Ile) and rs746462745 c.164C>G p.(Thr55Ser), had a severe 8mo cholestatic episode in early infancy, with persisting hyperbilirubinemia and fibrosis on imaging studies at 17y. No patient had skeletal malformations.

**Conclusion:**

Our findings suggest association of *IFT172* variants with non-syndromic cholestatic liver disease.

## Introduction

Genetic defects contribute to intrahepatic cholestasis in both children and adults [1]. Using a phenotype-based candidate gene approach, patients can be successfully diagnosed by Sanger sequencing; nonetheless, sequencing of a 66-gene cholestasis panel designed by EGL Genetics in 2017 increased the chance of diagnostic success in infants, children, and young adults, replacing single-gene analysis as a primary diagnostic tool [2]. The Mayo Clinic Laboratories currently offer testing for 112 “cholestasis genes” (test ID: CHLGP); even this is likely not exhaustive.

Limitations imposed by panel size can be overcome by focused-exome sequencing (FES) using capture platforms that target ~5000 genes mutated in Mendelian genetic diseases (Mendeliome) [3] or by whole-exome sequencing (WES). WES is still considered a discovery tool rather than a standard diagnostic procedure although its usefulness has been repeatedly proven in paediatric patients and recently also in adults [4]. The main advantage of WES, coverage of most expressed transcripts, opens the way to unexpected diagnoses, with identification of novel genetic disorders [5–10] and gene-phenotype relationships such as liver manifestations of known extrahepatic diseases without or with secondary liver injury. Whereas the main disadvantageous features of WES–need for excessive data processing and higher cost–have already been overcome, one should keep in mind that, as with FES or panel sequencing, even in WES methodology limitations inhere: Not all exons may be captured, sensitivity for structural variations is low, and WES does not include most non-coding regions [11].

In this study we explored the contribution of WES to molecular diagnosis in 51 patients with intrahepatic cholestasis.

## Patients and methods

### Patients

Patients with cholestatic liver disease or a history of cholestasis of unknown aetiology documented by detailed medical reports from the referring centre were eligible. Cholestasis was defined as conjugated hyperbilirubinemia accompanied by bilirubinostasis in hepatocytes, canaliculi and Kupffer cells in acinar zone 3, and / or by ductular reaction in acinar zone 1 on histopathologic study, or by elevated serum bile salt level exceeding 10 μmol/l. Patients with extrahepatic bile duct obstruction or autoimmune hepatobiliary disease and those fed only parenterally were excluded. Phenotyping was based on clinical reports and accessible histopathology reports provided by the referring centres (see S1 File). The authors had access to information that could identify individual participants during or after data collection. Where possible, samples from first-degree relatives were obtained for segregation studies. Composed identifiers (IDs) used for index subjects in the results section and tables indicate gender (M—male, F–female), patient age in years at specimen receipt, origin (CE—Central Europe, RO–Roma, PA–Australian Pacific, EA–East Asia, ME–Middle East), and unique number indicating order of specimen receipt. All 18 enrolled adult patients, legal guardians of all 33 enrolled children and all 58 available first-degree relatives who provided DNA samples signed written informed consent for genetic testing approved by the relevant institutional human research committees. The study protocol conforms to the ethical guidelines of the 1975 Declaration of Helsinki as reflected in *a priori* approval by the institution’s human research committee of IKEM and Thomayer Hospital in Prague, Czech Republic (Docket No. 17-06-18, date June 28, 2017, study period from July 2017 till December 2022).

**Fig 1:**
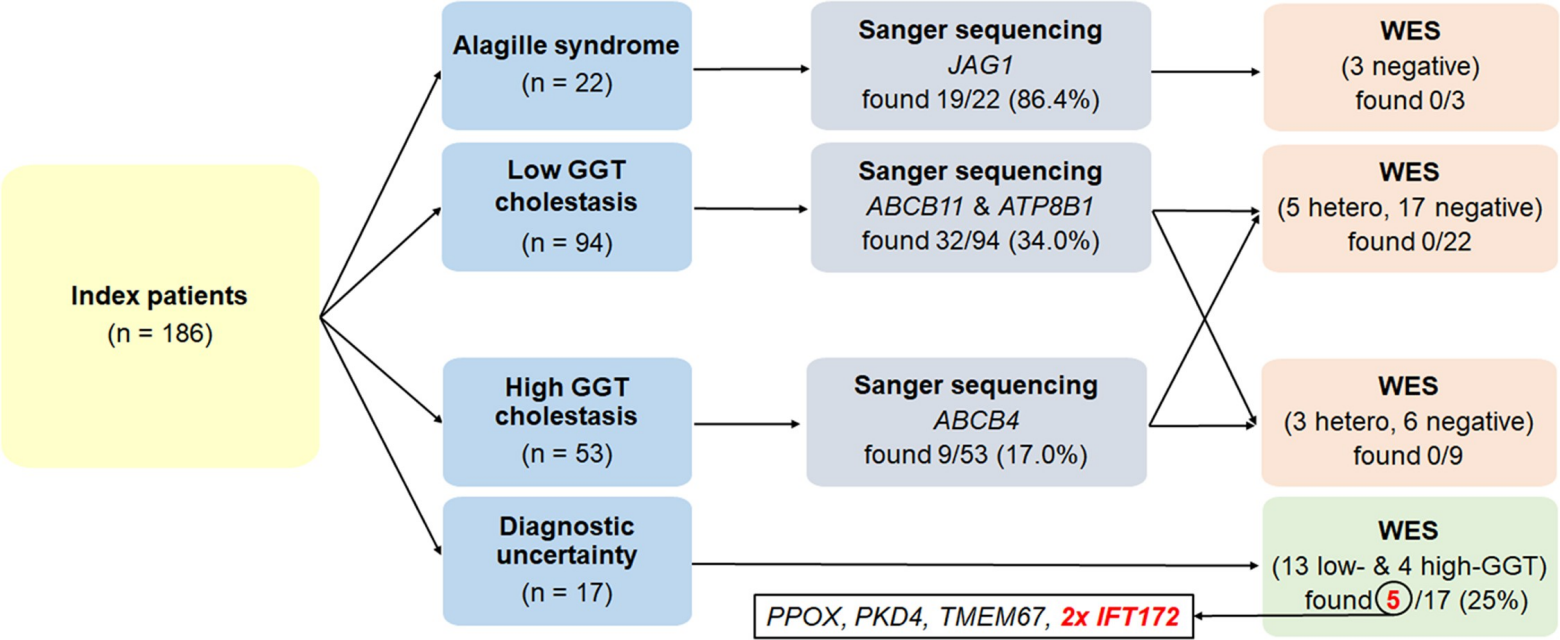
Patient enrolment and sample processing diagramme. Using a phenotype-based candidate gene approach, 169 index patients were screened by Sanger sequencing for variants in *JAG1* (n = 22), *ABCB11* & *ATP8B1* (n = 94), and *ABCB4* (n = 53). The remaining 17 index patients with diagnostic uncertainty were directly examined by WES. Nineteen of the 22 clinically diagnosed Alagille syndrome patients carried monoallelic P, LP or VUS variants in *JAG1* (S3A Table). Forty-one of the 147 patients with non-syndromic cholestasis were homozygotes or compound heterozygotes for P/LP/VUS variants in either *ABCB11* or *ATP8B1* (32 patients with low-GGT cholestasis, S3B Table) or in *ABCB4* (9 patients with high-GTT cholestasis, S3C Table). The 60 patients carrying monoallelic variants in *JAG1* or biallelic variants in *ABCB11*, *ATP8B1* or *ABCB4* were excluded.

Thirty-four enrolled index patients (samples collected between December 2006 and February 2021) represented a subset of 109 patients negatively screened by Sanger sequencing since June 2002 (Fig 1). Most of the remaining 75 patients were lost to clinical follow-up; in a few the diagnosis was re-classified as non-genetic disease. Seventeen index patients with clinically uncertain diagnosis due to various extrahepatic comorbidities such as kidney disease, osteogenesis imperfecta, cutaneous porphyria, orofacial cleft or episodic fever, were examined directly by WES between October 2018 and November 2021.

### Histologic and immunohistologic study

Percutaneous liver-biopsy specimens were fixed in 4% paraformaldehyde and routinely processed for histological examination. Paraffin sections cut at 4 μm were stained with haematoxylin and eosin, Weigert-van Gieson with resorcin-fuchsin, periodic acid-Schiff reaction (PAS) with and without diastase pre-digestion, orcein, and Perls’ reaction. For immunohistochemical analysis, sections were incubated with anti-cytokeratin 7 antibody at a dilution of 1:100, anti-MDR3/ABCB4 rabbit polyclonal antibody at a dilution of 1:20, and anti-BSEP/ABCB11 rabbit polyclonal antibody at a dilution of 1:200 (details, S1 Table). Primary antibodies were detected by ultraView Universal DAB Detection Kit (Roche Tissue Diagnostics [formerly Ventana Medical Systems], Basel, Switzerland).

#### DNA isolation

Genomic DNA was extracted from peripheral blood by QIAamp DNA Blood Kit (Qiagen, Hilden, Germany) with standard procedures.

### Whole-exome sequencing

After mechanical fragmentation, exome libraries were constructed according to the manufacturer’s standard protocol with TruSeq® Exome Kit (Illumina, San Diego, CA). WES was accomplished using the Illumina NextSeq 500 platform. Alignment to the reference sequence (hg19 build) and variant calling was performed by Qiagen CLC Genomics Workbench 12 (https://digitalinsights.qiagen.com/). Identified variants were selected for annotation and interpretation using a 5% minor allele frequency (MAF) threshold taken from the gnomAD database. IGV Viewer [12] was used to display the data. Exome sequencing data are deposited in the General University Hospital in Prague and National Centre for Medical Genomics databases. Access conditioned by permission from the General University Hospital Ethics Committee.

### Sanger sequencing

DNA sequencing of gel-purified PCR products (primer list, S2 Table) was performed by 3.1 Dye Terminator cycle sequencing kit and 3130 Genetic Analyzer electrophoresis (both Thermo Fisher Scientific, Waltham, MA).

### Variant classification

Variants were classified according to American College of Medical Genetics and Genomics [ACMG] professional guidelines [13] using the VARSOME tool [14], published literature, variant databases NCBI ClinVar & Human Gene Mutation Database (HGMD), and population frequency information compiled in NCBI dbSNP. Sequence variations were assigned among the 5 categories benign, likely benign, variant of unknown significance (VUS), likely pathogenic (LP), or pathogenic (P). We characterized carriage of one P or LP variant as diagnostic for autosomal-dominant diseases and carriage of P/P, P/LP, or LP/LP variants as diagnostic for autosomal-recessive diseases.

### Detection of copy number variations (CNV) and homozygosity regions

The command-line software toolkit CNVkit version 0.9.6CNV was used for CNV analysis of exome-sequencing data. Validation was performed by array comparative genome hybridization (CGH) using the commercially available oligonucleotide platforms GenetiSure Cyto 4x180K and SurePrint G3 CGH+SNP 4x180K (both Agilent Technologies, Santa Clara, CA). The latter platform was also used for detection of homozygosity regions. Data were analysed by CytoGenomics software version 5.1.2.1 (Agilent Technologies). UCSC Genome Browser, DECIPHER, NCBI ClinVar, OMIM, and DGV databases were used for assessment of clinical impacts of deletions and duplications.

### Long range PCR and long-range amplicon single strand sequencing

A region containing the variants of interest in *IFT172* was directly amplified from genomic DNA using TaKaRa LA Taq DNA Polymerase with 10x LA PCR Buffer II (TaKaRa, Mountain View, CA) with a two-step PCR protocol (primers, S2 Table). Initial denaturation at 94°C for 1min was followed by 30 cycles of 98°C for 10s denaturation and 64.1°C for 22min and 30s elongation. Final elongation was performed by incubation at 72°C for 10min. Product length was 22,800 bp. LR-PCR products were purified using SPRI magnetic beads (Beckman Coulter Life Sciences, Brea, CA) according to the manufacturer’s protocol, with 2μl used for Qubit 2.0 Fluorometric Quantitation (Beckman Coulter Life Sciences) dsDNA high sensitivity assay. Purified samples were sequenced by Pacific Biosciences Sequel system (PacBio, Menlo Park, CA) according to manufacturer’s protocol using SMRTbell Express Template Kit 2.0 and Sequel Sequencing Kit 3.0. To obtain highly accurate reads, Circular Consensus Sequence (CCS) analysis was performed using Pacific Biosciences SMRT Link (v6.0). CCS reads were aligned to human reference genome (hg19) using Minimap2 v. 2.24 [15], sorted with SAMtools and visualized in IGV browser.

### Nasopharyngeal swab mRNA analysis

Total RNA was isolated from nasopharyngeal swabs using viRNAtrap and magnetic beads from GeneSpector (Prague, Czech Republic). RNA libraries were prepared using KAPA RNA HyperPrep Kit with RiboErase (Roche, Basel, Switzerland) according to manufacturer’s instructions. RNA sequencing (2x100 paired-end reads) was performed in 3 patients and family members using the NovaSeq 6000 system (Illumina, San Diego, CA) at the National Center for Medical Genomics in Prague. The resulting files in FASTQ format were subjected to quality control and trimmed using Atropos v.1.128 [16]. Gene-level abundances were estimated using Salmon v.1.3 [17] with Ensembl based annotation package *EnsDb*.*Hsapiens*.*v75*. Normalization and differential expression analyses were performed within the DESeq2 R package [18].

Coding DNA was transcribed from RNA isolated from nasopharyngeal swab using SuperScript™ IV Reverse Transcriptase (ThermoFisher), oligo dT 23VN and random hexamers following manufacturers protocol. A region encompassing *F11R* exons 1–10 was amplified using Phusion Hot Start Flex DNA Polymerase (Thermo Fisher Scientific) with a three step PCR protocol. The 1,052 bp product was purified using SPRI magnetic beads (Beckman Coulter Life Sciences, Brea, CA) and the region of interest was sequenced (primers, S2 Table).

## Results

WES was performed in 51 clinically unrelated index subjects and in 58 available family members. Demographic characteristics and laboratory phenotype of the enrolled index subjects are presented in Table 1.

**Table 1:** Demographic characteristics and phenotypes of enrolled index subjects.

Age at referral (years)	Low-GGT cholestasis		High-GGT cholestasis	
	male	female	male	female
<2	4	3	4	0
2–18	13	2	4	3
≥19	3	8	3	4

Low GGT means serum GGT activity ≤ 1.04 μmol/l for the age category 7wk– 52wk, ≤ 0.39 μmol/l for children aged 1y – 15y, ≤ 0.84 μmol/l for males aged > 15y and ≤ 0.64 μmol/l for females aged > 15y.

In 26 index subjects (18 children at referral), initial Sanger sequencing had found no aetiologic variants in *JAG1* (OMIM 601920), *ABCB11* (OMIM 603201), *ATP8B1* (OMIM 602397), or *ABCB4* (OMIM 171060). In 8 single heterozygotes (5 children at referral) for P/LP variants in *ABCB11*, *ATP8B1* (S4A and S4B Table) or *ABCB4* (S4A and S4C Table), clinical findings (with disease) were discordant with heterozygosity for just one variant. WES did not reveal genetic causes of liver disease in these 34 patients; however, among the remaining 17 families (10 index patients were children at referral), unexpected molecular findings potentially relevant for genetic liver disease were obtained in 6 patients (Table 2).

**Table 2:** Candidate causative pathogenic / likely pathogenic variants and variants of unknown significance compatible with patient phenotype and type of inheritance.

Patient ID	Clin. dx.	P / LP variants	VUS
F48CE680	PCT, low-GGT cholestasis	*PPOX* rs774663053 c.397G>T p.(Glu133*), CM981616, HET	not found
F9CE723 (+ parents)	low-GGT PFIC	***PKHD1* rs760222236 c.8870T>C p.(Ile2957Thr),** CM020500, HET, maternal	***PKHD1* NM_138694.4 c.10768A>T p.(Ile3590Phe),** HET, paternal
M18CE172 (+ parents)	low-GGT PFIC	***TMEM67* rs201893408 c.1843T>C p.(Cys615Ser)**, CM094694, HOM, both unaffected parents HET	not found
M26RO684	glycogenosis, high-GGT PFIC	not found	***IFT172* rs780205001 c.167A>C p.(Lys56Thr)**^a^, HOM
M14CE762	low-GGT PFIC	*IFT172* NM_015662.3 c.2070del p.(Met690Ilefs*11), HET	***IFT172* rs776310391 c.157T>A p.(Phe53Ile)**, HET ***IFT172* rs746462745 c.164C>G p.(Thr55Ser)**, HET
F1RO453 (+ parents + 2 sisters)	low-GGT PFIC	***F11R* NM_016946.6 c.65-2A>T**, HOM, both unaffected parents HET, unaffected sisters HOM and HET	not found

Variants are classified according to the ACMG criteria [13] were valid in week 20, 2023. Clin. dx.–clinical diagnosis. GGT–gamma-glutamyl transferase, PFIC–progressive familial intrahepatic cholestasis, PCT–porphyria cutanea tarda, rs–dbSNP accession number, CM–HGMD accession number, HET–heterozygous state, HOM–homozygous state.

^a^The Association of Molecular Pathology classification of the marked variant is LP.

In 3 of these patients (ID F48CE680, F9CE723, and M18CE172), molecular diagnoses respectively of variegate porphyria (VP, OMIM #176200), polycystic kidney disease with or without polycystic liver disease 4 (PKD4, OMIM #263200), and nephronophthisis 11 (NPHP11, OMIM #613550, ref. [19]) were established. Two other index subjects harboured biallelic variants in *IFT172* (OMIM 607386):

### Patient 1 with biallelic variants in *IFT172*

**Fig 2:**
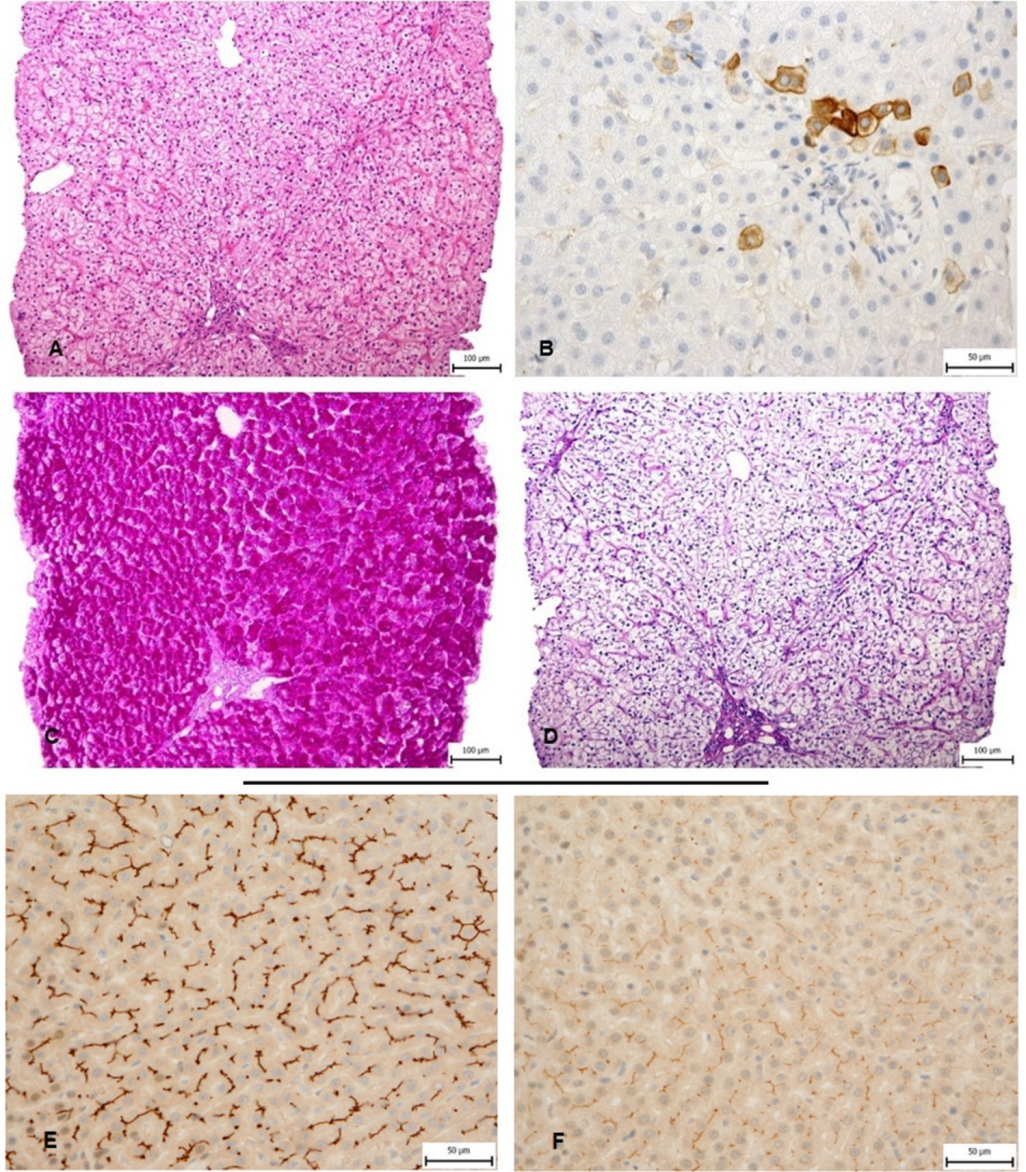
Photomicrographs, liver of patient homozygous for variant c.167A>C p.(Lys56Thr) in *IFT172* (index patient ID M26RO684, aged 4y). (**A**) Preserved lobular architecture with enlarged and pale hepatocytes (haematoxylin and eosin, original magnification 200x). (**B**) Interlobular bile duct deficiency with aberrant CK7 expression in some periportal hepatocytes (anti-CK7 immunostaining—CK7-expressing cells highlighted in brown / negative background blue, original magnification 400x). (**C**) Glycogen accumulation in hepatocytes with diffuse PAS reactivity, (**D**) lacking after diastase pre-digestion (original magnification 200x both). (**E**) Strong diffuse expression of ABCB11 on canalicular membranes of hepatocytes (anti-ABCB11 immunostaining, original magnification 400x). (**F**) Focally attenuated expression of ABCB4 on canalicular membranes of hepatocytes (anti-ABCB4 immunostaining, original magnification 400x).

A Roma man (ID M26RO684, Table 2) aged 26y, living with foster parents and without available birth-family members, had chronic liver disease with hepatomegaly since early childhood. Histopathologic study of a liver-biopsy specimen obtained at age 4y found preserved hepatic lobular and vascular architecture with well-glycogenated hepatocytes and trivial steatosis; fibrosis, inflammation, and accumulations of bile pigment were not seen (Fig 2).

Glycogen storage disease (GSD) was suspected and the patient was repeatedly examined by a specialist in genetic metabolic diseases. Activities of phosphorylase in leukocytes and of phosphorylase kinase and glycogen debrancher enzyme in erythrocytes investigated at age 9y were within normal ranges, leaving mild forms of GSD types I or III still under consideration. Alanine aminotransferase (ALT) and aspartate aminotransferase (AST) activities were elevated (ALT 1.5x upper limit of normal [ULN], AST 2x ULN) without abnormal values for other biomarkers (serum bilirubin, GGT, blood glucose, lactate and lipid profile, renal-function indicators, and erythrocyte glycogen content) at age 11y. Dietary measures were introduced and metabolic-disease specialists supervised the patient till age 25y, when end-stage kidney disease of unknown aetiology requiring initiation of haemodialysis maintenance was diagnosed. Evaluation for renal transplant took place at the Institute of Clinical and Experimental Medicine.

On admission, apart from severely elevated urea and creatinine values, mildly increased serum AST activity (2.7x ULN) and severely increased cholestasis-biomarker enzyme activities (alkaline phosphatase 6.5x ULN, GGT 11.3x ULN) were observed, without hyperbilirubinemia. Ultrasonography revealed bilateral renal atrophy without abnormalities of liver or spleen. Percutaneous liver biopsy was repeated. The light-microscopy finding of mild hepatocellular glycogen deposition was unchanged. In addition, transfusional siderosis and lack of interlobular bile ducts in 5 of 14 portal tracts accompanied by mild features of chronic cholestasis were observed. Portal-tract fibrosis, hamartomatous or cystic bile-duct profiles, and hypoplasia of portal-vein radicles were absent; criteria for diagnosis of ductal plate malformation were not met. Canalicular expression of the homologues BSEP/ABCB11 and MDR3/ABCB4 was preserved, but for the latter was strikingly less prominent than for the former, suggesting *ABCB4* disease. WES was requested to aid in diagnosis.

**Fig 3:**
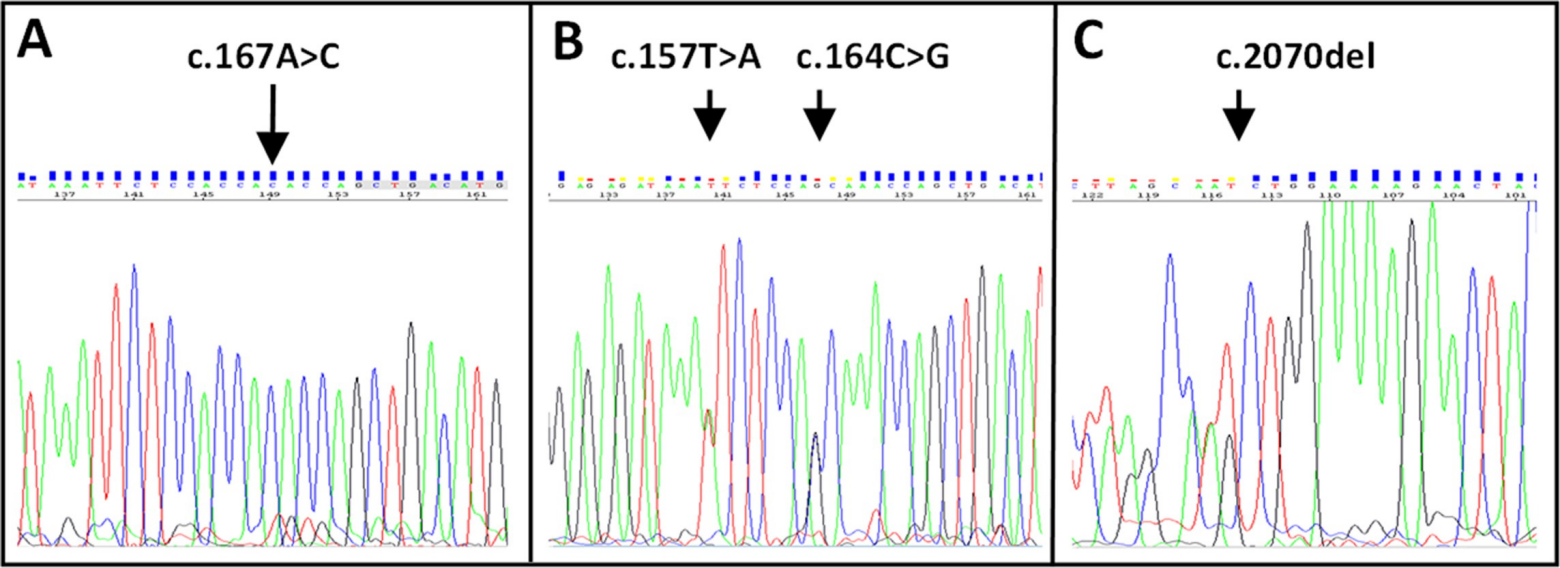
Validation electropherograms of *IFT172* partial genomic DNA sequence of exon 2 from index subjects M26RO684 (**A**) and M14CE762 (**B**), and exon 20 from index subject M14CE762 (**C**).

P/LP variants were detected among neither 66 neonatal/adult cholestasis genes [2] nor 21 genes mutated in GSD [20]. Bioinformatics analysis revealed a homozygous variant, rs780205001 c.167A>C (p.Lys56Thr), in *IFT172*, validated by Sanger sequencing (Fig 3A). The variant, rare in population databases (ExAC 0.0000119, GnomAD_exome 0.0000084, ALFA 0.005%) and not found in 110 unrelated Roma individuals, has not been identifiably reported in *IFT172*-related conditions. The lysine residue is highly conserved and a moderate physicochemical difference between lysine and threonine exists. The available evidence is currently insufficient to determine the role of this variant in disease. Therefore, the variant is classified as VUS in the NCBI ClinVar database (acc. no. VCV001015325.2). However, using the ACMG classification, Varsome suggested “warm” VUS (ACMG/AMP = PS4_supp (1p)+ PM2_supp (1p) + PM3_supp (0.5p)+ PP3 (1p)+ PP4 (1p)+ BP1 (-1p) = 3.5points >>VUS (tepid/warm)) and the prediction programmes PredictSNP (all MAPP, PhD-SNP, PolyPhen, SIFT and SNAP) and MutationTaster suggested likely pathogenicity. Accordingly, advanced modelling of protein sequence and biophysical properties (such as structural, functional, and spatial information, amino acid conservation, physicochemical variation, residue mobility, and thermodynamic stability) performed at Invitae (San Francisco, CA) indicates that this missense variant is expected to disrupt IFT172 protein function.

RNAseq of mRNA isolated from nasopharyngeal swabs confirmed that the variant NM_015662.3 r.257A>C was present in all reads from the *IFT172* transcripts, without impact on *IFT172* mRNA expression level, which was within population expression boundaries. No abnormal alternative splicing event in *IFT172* was observed. The impact of the variant on IFT172 protein expression or subcellular localisation in the liver biopsy specimen could not be assessed due to the lack of a suitable antibody.

Parental unavailability precluded confirmation of homozygosity for the *IFT172* variant by analysis of parental DNA samples. To distinguish between true homozygosity and hemizygosity for *IFT172* c.167A>C, CNV analysis focused on the *IFT172* locus ([GRCh37]: chr2: 27667244–27712610) was repeated. Read number did not fall. Moreover, WES data identified the variant as located in a 24.03Mb homozygosity region ([GRCh37], chr2:20440321–44471409). To validate this, we performed genomic hybridisation on two CGH arrays. In line with exomic data, no copy-number changes in the *IFT172* region (2p23.3) or other CNV(s) explaining the proband’s phenotype were identified. The CGH SNP array detected loss of heterozygosity of autosomal regions (total range 322Mb; 11.2% of haploid autosomal genome). Coefficient of consanguinity (*f*) of the proband’s biological parents was 1/4. We also confirmed that *IFT172* lay in a 27.2Mb homozygosity region (arr[GRCh37] 2p23.3(19824727_47021455)x2 hmz).

Within 1y the patient´s body mass index (BMI) recovered from 27.6 to 33 (obesity grade I). At age 26y the patient was listed for kidney transplantation, performed at age 27y.

The finding of a homozygous c.167A>C p.(Lys56Thr) “warm” VUS in *IFT172* suggested short-rib thoracic dysplasia 10 (SRTD10, OMIM #615630) or Bardet-Biedl syndrome 20 (OMIM #619471). Further physical examination and roentgenograms of the chest, hips, legs, and feet revealed no bone malformations. Nor did ophthalmologic examination identify retinitis pigmentosa, typical in IFT172 deficiency (S1 Fig). Best corrected visual acuity was bilaterally normal (1.0 Snellen decimal values), as was intraocular pressure (19mmHg OD, 15mmHg OS). The patient interestingly had an abnormally thin retinal ganglion cell layer, documented using spectral domain optical coherence tomography (Spectralis, Heidelberg Engineering, Heidelberg, Germany).

### Patient 2 with biallelic variants in *IFT172*

A caucasian boy (ID M14CE762, Table 2) born to unaffected parents, manifested severe icterus, hepatosplenomegaly, and coagulopathy aged 2mo, with hyperbilirubinemia (total bilirubin 18x ULN, direct bilirubin 27.3x ULN), abnormal hepatobiliary-injury biomarker values (AST 8.9x ULN, ALT 2.8x ULN), and hypercholanemia (30x ULN). GGT activity was within normal range. Light microscopy of a liver-biopsy specimen found giant-cell change of hepatocytes with cholestasis and fibrosis; steatosis and glycogen accumulation were absent. By age 10mo hyperbilirubinemia was nearly resolved; however, it has persisted (age 18y, total and direct bilirubin 1.4x and 1.1x ULN respectively) and findings on elastography (liver stiffness 9 kPa) indicate liver fibrosis and steatosis. The patient´s BMI is 20.6. Aside from nystagmus, physical-examination and laboratory findings are otherwise unremarkable, with in particular no skeletal malformations and no signs of kidney disease. He carries 3 variants in *IFT172*: On one allele he harbours the novel deletion c.2070del p.(Met690Ilefs*11, Fig 3C), classified as likely pathogenic (PVS1+PM2 = 9 points), and on the other (*i*.*e*., in *trans*) 2 single-nucleotide substitutions, rs776310391 c.157T>A p.(Phe53Ile) and rs746462745 c.164C>G p.(Thr55Ser) (Fig 3B), both classified as VUS according the ACMG guidelines and likely pathogenic based on the Association for Molecular Pathology criteria (c.157T>A: PM2_supp (1p) + PM3_supp (1p) +PP3 (1p) = 3 points >>VUS (tepid), c.164C>G: PM2_supp (1p) + PM3_supp (1p) + BP1 (-1p) + BP4 (-1p) = 0 points >>>VUS (ice cold)). Since both parents refused genetic testing, relative position of the variants was assessed by single strand sequencing of long-range PCR products encompassing all 3 mutated loci.

### Carrier of biallelic variant in *F11R*

**Fig 4:**
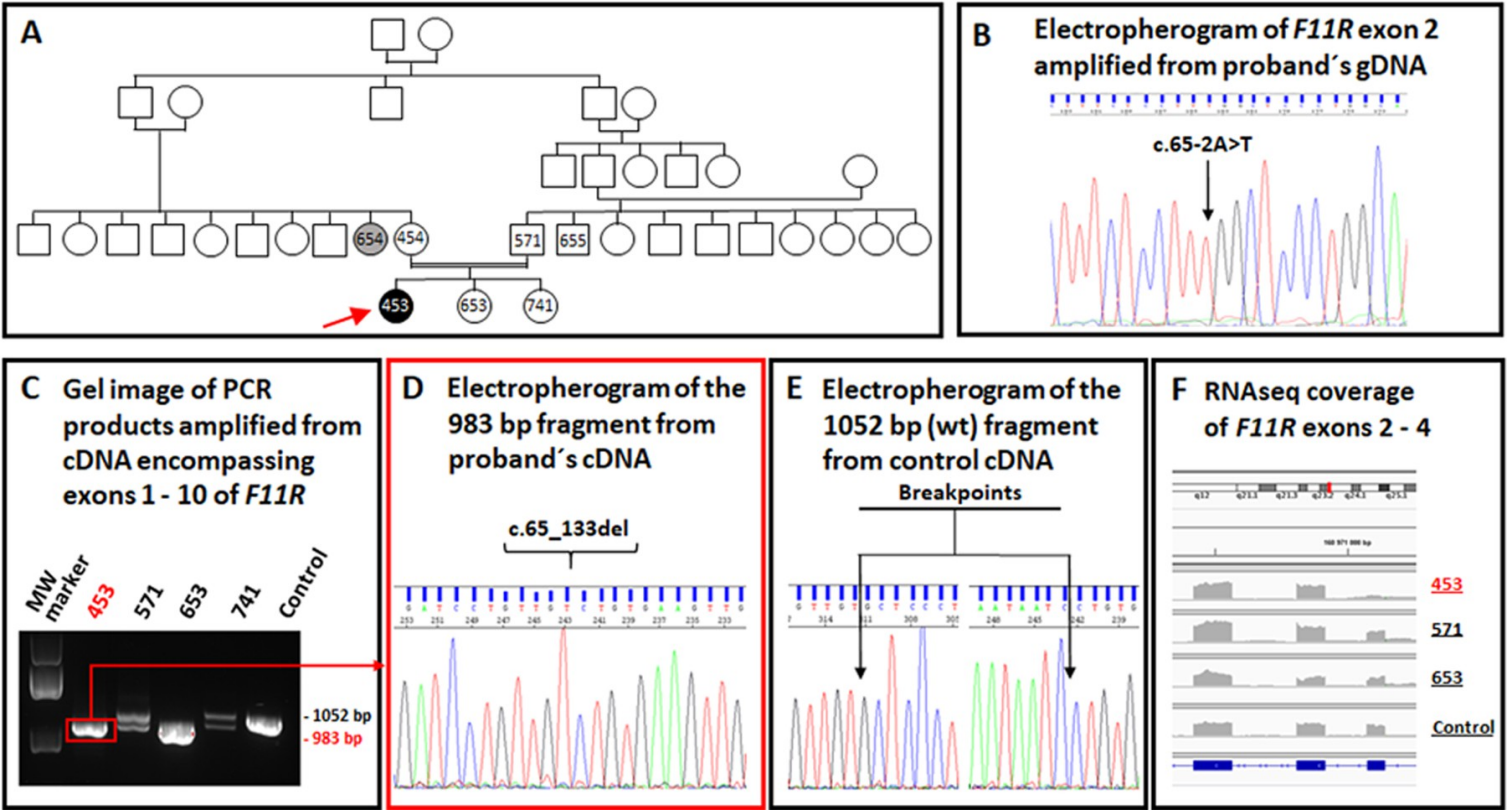
Molecular findings, family with JAM1 deficiency (index patient ID F1RO453). (**A**) Pedigree; arrow, index patient. (**B**) Sanger-sequencing electropherogram of *F11R* exon 2 amplified from the index patient´s gDNA. Arrow, homozygous *F11R* c.65-2A>T splice-site variant. (**C**) Agarose-gel electropherogram of PCR products encompassing exons 1–10 amplified from nasopharyngeal swabs *F11R* cDNA. (**D**) Sanger-sequencing electropherogram of the 983-bp fragment amplified from nasopharyngeal-swab cDNA of proband. Arrow, skipped exon 2. (**E**) Sanger-sequencing electropherogram of the 1052-bp fragment amplified from control nasopharyngeal-swab cDNA (wild-type sequence). Arrows, breakpoints. (**F**) Graphical representation of *F11R* exons 2, 3, and 4 coverage by RNAseq of nasopharyngeal-swab mRNA in IGV Viewer.

Finally, in a one-year-old (now 8y) Roma girl (ID F1RO453) with cholestatic cirrhosis, WES revealed a yet unreported homozygous splice-site variant, c.65-2A>T, in *F11R* (OMIM 605721), encoding the tight junction protein JAM1 (Figs 4 and S2).

Both unaffected parents were asymptomatic heterozygotes for the *F11R* c.65-2A>T variant.

Since no liver-biopsy specimen suitable for mRNA analysis was available, we analyzed nasopharyngeal-swab mRNA by RNAseq. Apart from the expected in-frame deletion of 69 base pairs (NM_016946.6 r.144_212del) caused by skipping of exon 2 (confirmed by Sanger sequencing of *F11R* cDNA), RNAseq revealed no P/LP variants. The *F11R* variant is predicted to replace 24 amino-acid residues by serine (p.Cys22_Pro45delinsSer). Such an indel should impair the function of the altered protein, if expressed, by removing the motif Asn43-Asn44-Pro45, involved in *trans*-homophilic interaction of JAM1 *cis*-dimers [21]. Unfortunately, clinical pathogenicity of the variant is uncertain: The same homozygous variant exists in the proband´s sister, aged 7y and unaffected at this writing.

### Extrahepatic diseases

Apart from the patients described above, other genetic diagnoses were suggested in another three probands (Table 3). These include osteogenesis imperfecta caused by a recently reported variant in *COL1A2* (OMIM 120160) [22], familial periodic fever associated with a known variant in *TNFRSF1A* (OMIM 191190), and a combination of orofacial cleft with growth and mental retardation contributed by variants in *TP63* (OMIM 603273), *COL2A1* (OMIM 120140), and *ZNF423* (OMIM 604557) [23].

**Table 3:** Variants supporting other genetic diagnoses.

Patient ID	Clin. dx.	P / LP variants	Variants of unknown significance
F57CE614 (+ mother)	low-GGT PFIC	***COL1A2* rs67525025 c.767G>T p.(Gly256Val)**, CM062547, HET, non-maternal	not found
F6CE561 (+ parents)	high-GGT PFIC	***TNFRSF1A* rs4149584 c.362G>A p.(Arg121Gln)**, CM012483, HET, maternal	not found
M3RO646 (+ parents)	high-GGT PFIC	***TP63* rs768752805 c.799G>A p.(Val267Ile)**, CM1812821, HET, paternal ***ZNF423* rs548986682 c.3370G>A p.(Glu1124Lys)**, CM1512351, HET, maternal	***COL2A1* rs201675352 c.410G>A p.(Arg137His)**, CM160186, HET, paternal

Variants are classified according to the ACMG criteria [13] valid in week 20, 2023. Clin. dx.–clinical diagnosis. GGT–gamma-glutamyl transferase, PFIC–progressive familial intrahepatic cholestasis, rs–dbSNP accession number, CM–HGMD accession number, HET–heterozygous state, HOM–homozygous state.

## Discussion

In our cohort of 51 enrolled index patients, 34 of whom were extensively pre-screened by targeted gene approach, WES revealed unexpected findings in just 6 patients and other or additional genetic diagnoses in another 3 subjects. Multiple reasons for low efficiency can be adduced. Technical limitations of WES have been touched on; perhaps more important are difficulties with interpretation of variant pathogenicity. Sequencing of individual exomes provides lists of VUS never assessed experimentally. ACMG classification of individual variants is partially based on indirect indicators and *in silico* predictions–these in some situations may be misleading. That ClinVar classifications of numerous variants are equivocal, with several classifications of the same variant proposed, thus is unsurprising.

Concerning the capacity of WES to reveal rare unexpected principal and additional diagnoses, WES is obviously superior to sequencing a single gene or a disease gene panel. In the patient with jaundice associated with cutaneous porphyria (ID F48CE680), WES established the molecular diagnosis of variegate porphyria. This experience illustrates that diagnosis of disorders manifesting as cutaneous porphyria, with cholestasis and a negative family history but without a personal history of neurovisceral attacks, is scarcely possible without molecular-genetic studies.

Whereas congenital hepatic fibrosis was a clinical consideration in the second patient (ID F9CE723) despite the absence of cystic biliary-tract and renal disease detected after establishing the genetic diagnosis, ciliopathy gene variants were unexpected in the other three patients. In two, renal disease did not meet diagnostic criteria for nephronophthisis [19] before renal failure developed. Indeed, associations between liver disease and ciliopathy syndromes, including PKD4 and NPHP11, are published (review, Diamond *et al*. [24]).

The phenotype of IFT172 disease–short rib thoracic dysplasia 10 without polydactylv–varies greatly. It encompasses asphyxiating thoracic dysplasia (Jeune syndrome) [25], Mainzer-Saldino syndrome [25], isolated retinal degeneration [26], Bardet-Biedl syndrome [26, 27], and oral-facial-digital syndrome [28]. Some patients with Jeune or Meinzer-Saldino syndrome have nephronophthisis designated as NPHP17. Liver disease includes bile-duct abnormalities with cystic dilatation and periportal fibrosis. Although liver abnormalities may attract medical attention as the first sign of Jeune syndrome [29], they are considered minor and inconsistent ciliopathy features encountered in Jeune syndrome even with only mild skeletal malformations [30].

Non-syndromic cholestatic liver injury in IFT172 disease without skeletal malformations–and without bile-duct abnormalities–has never been identifiably reported. Our observation of 2 unrelated carriers of biallelic IFT172 variants with unexplained primary cholestatic liver disease, strengthened in the older patient (ID M26RO684) by adult-onset nephronophthisis, location of the homozygous *IFT172* variant in a large homozygosity region inherited from consanguineous parents (cousins) and absence of candidate variants in the other 18 nephronophthisis genes, suggests association. Reduced retinal ganglion cell layer thickness observed in the older patient can be a sign of early pre-perimetric glaucoma [31]; however, given the age of the patient and normal intraocular pressure, a link with *IFT172* cannot be excluded.

What diagnosis to assign to the sixth patient, who harbours a homozygous splice-site variant in *F11R*, remains unclear. F11R deficiency has never been identifiably reported in humans. Since *F11r* ^-/-^ mice are highly susceptible to liver injury [32–34] and since deficiency of other tight junction proteins, namely tight junction protein 2 and claudin-1, is associated with cholestatic liver disease [35, 36], to explain the proband´s phenotype as a consequence of JAM1 deficiency tempted us. That the proband´s unaffected sister carries the same *F11R* variant in homozygous state, however, casts doubt on the association. One might invoke incomplete penetrance driven by unknown genetic or environmental factors to explain this phenotypic variation, but studies in more families with JAM1 deficiency are essential to settle the matter.

An important favourable characteristic of genomic analysis technologies in general is that they are non-invasive. The clinical utility of biopsy in genetic liver diseases is limited [1]; in addition, patients or their parents may refuse liver biopsy, considering it unacceptably risky. Broader availability of RNAseq has opened the way to pathogenicity studies of candidate variants in liver-disease–causing genes expressed in extrahepatic epithelial tissues such as those accessible by nasopharyngeal swab (see S5 Table). As demonstrated in our patients with pathogenic variants in *IFT172* and *F11R*, nasopharyngeal-swab mRNA analysis can be more informative than can immunohistologic assessment of expression of a limited panel of proteins in liver-biopsy specimens. Based on this experience, we believe that combined analysis of the whole exome and of nasopharyngeal-swab mRNA will make up for loss of some information hitherto gained in genetic liver diseases via histopathologic study.

In conclusion, we confirm the clinical utility of WES in patients with suspected genetic cholestasis. Combining WES with nasopharyngeal-swab mRNA analysis improves interpretation of WES data. Our findings suggest association of *IFT172* variants with primary cholestatic liver disease which awaits confirmation by additional similar observations.

## Supporting information

S1 File List of referring centres. (DOCX)

S1 Table Antibodies. (DOCX)

S2 Table Sequence based reagents. (DOCX)

S3 Table Variants detected by initial Sanger sequencing in 60 excluded patients. (DOCX)

S4 Table Clinical diagnosis and variants found by initial Sanger sequencing in 34 enrolled patients. (DOCX)

S5 Table Nasopharyngeal swab expression of cholestasis and ciliopathy genes. (DOCX)

S1 Fig Retinal findings in patient homozygous for the *IFT172* c.167A>C variant. (PDF)

S2 Fig Molecular findings, family with JAM1 deficiency. (PDF)

S1 Checklist STROBE statement—checklist of items that should be included in reports of observational studies. (DOCX)

## Abbreviations

ABC: ATP-binding cassette
ACMG: American College of Medical Genetics and Genomics
ALT: alanine aminotransferase
AST: aspartate aminotransferase
BMI: body mass index
BSEP: bile-salt export pump
CCS: circular consensus sequence
CGH: comparative genomic hybridization
COL: collagen; FES, focused-exome sequencing
GGT: gamma-glutamyl transferase
GSD: glycogen storage disease
ID: identifier
IFT: intraflagellar transport
JAG: jagged canonical notch ligand
JAM: junctional adhesion molecule
LP: likely pathogenic
MAF: minor allele frequency
MDR: multiple drug resistance
NCBI: National Centre for Biotechnology Information
NPHP: nephronophthisis
OMIM: Online Mendelian Inheritance in Man
P: pathogenic
PAS: periodic acid-Schiff
PKHD: polycystic kidney and hepatic disease
PKD: polycystic kidney disease
PPOX: protoporphyrinogen oxidase
SNP: single nucleotide polymorphism
SRTD: short-rib thoracic dysplasia
TMEM: transmembrane protein
TNFRSF: tumour necrosis factor receptor superfamily
TP: tumour protein
ULN: upper limit of normal
VP: variegate porphyria
VUS: variant of unknown significance
WES: whole-exome sequencing
ZNF: zinc finger

## References

[1] Ibrahim SH, Kamath BM, Loomes KM, Karpen SJ. Cholestatic liver diseases of genetic etiology: Advances and controversies. Hepatology. 2022;75(6):1627–46. doi: 10.1002/hep.32437 35229330

[2] Karpen SJ, Kamath BM, Alexander JJ, Ichetovkin I, Rosenthal P, Sokol RJ, et al. Use of a comprehensive 66-gene cholestasis sequencing panel in 2171 cholestatic infants, Children, and Young Adults. J Pediatr Gastroenterol Nutr. 2021;72(5):654–60. doi: 10.1097/MPG.0000000000003094 33720099

[3] Pengelly RJ, Ward D, Hunt D, Mattocks C, Ennis S. Comparison of Mendeliome exome capture kits for use in clinical diagnostics. Sci Rep. 2020;10(1):3235. doi: 10.1038/s41598-020-60215-y 32094380 PMC7039898

[4] Hakim A, Zhang X, DeLisle A, Oral EA, Dykas D, Drzewiecki K, et al. Clinical utility of genomic analysis in adults with idiopathic liver disease. J Hepatol. 2019;70(6):1214–21. doi: 10.1016/j.jhep.2019.01.036 31000363 PMC6526061

[5] Luan W, Hao CZ, Li JQ, Wei Q, Gong JY, Qiu YL, et al. Biallelic loss-of-function ZFYVE19 mutations are associated with congenital hepatic fibrosis, sclerosing cholangiopathy and high-GGT cholestasis. J Med Genet. 2021;58(8):514–25. doi: 10.1136/jmedgenet-2019-106706 32737136

[6] Gao E, Cheema H, Waheed N, Mushtaq I, Erden N, Nelson-Williams C, et al. Organic solute transporter alpha deficiency: A disorder with cholestasis, liver fibrosis, and congenital diarrhea. Hepatology. 2020;71(5):1879–82. doi: 10.1002/hep.31087 31863603 PMC8577800

[7] Unlusoy Aksu A, Das SK, Nelson-Williams C, Jain D, Ozbay Hosnut F, Evirgen Sahin G, et al. Recessive mutations in KIF12 cause high gamma-glutamyltransferase cholestasis. Hepatol Commun. 2019;3(4):471–7. doi: 10.1002/hep4.1320 30976738 PMC6442693

[8] Maddirevula S, Alhebbi H, Alqahtani A, Algoufi T, Alsaif HS, Ibrahim N, et al. Identification of novel loci for pediatric cholestatic liver disease defined by KIF12, PPM1F, USP53, LSR, and WDR83OS pathogenic variants. Genet Med. 2019;21(5):1164–72. doi: 10.1038/s41436-018-0288-x 30250217

[9] Gonzales E, Taylor SA, Davit-Spraul A, Thebaut A, Thomassin N, Guettier C, et al. MYO5B mutations cause cholestasis with normal serum gamma-glutamyl transferase activity in children without microvillous inclusion disease. Hepatology. 2017;65(1):164–73. doi: 10.1002/hep.28779 27532546

[10] Gomez-Ospina N, Potter CJ, Xiao R, Manickam K, Kim MS, Kim KH, et al. Mutations in the nuclear bile acid receptor FXR cause progressive familial intrahepatic cholestasis. Nat Commun. 2016;7:10713. doi: 10.1038/ncomms10713 26888176 PMC4759630

[11] Burdick KJ, Cogan JD, Rives LC, Robertson AK, Koziura ME, Brokamp E, et al. Limitations of exome sequencing in detecting rare and undiagnosed diseases. Am J Med Genet A. 2020;182(6):1400–6. doi: 10.1002/ajmg.a.61558 32190976 PMC8057342

[12] Robinson JT, Thorvaldsdottir H, Winckler W, Guttman M, Lander ES, Getz G, et al. Integrative genomics viewer. Nat Biotechnol. 2011;29(1):24–6. doi: 10.1038/nbt.1754 21221095 PMC3346182

[13] Richards S, Aziz N, Bale S, Bick D, Das S, Gastier-Foster J, et al. Standards and guidelines for the interpretation of sequence variants: a joint consensus recommendation of the American College of Medical Genetics and Genomics and the Association for Molecular Pathology. Genet Med. 2015;17(5):405–24. doi: 10.1038/gim.2015.30 25741868 PMC4544753

[14] Kopanos C, Tsiolkas V, Kouris A, Chapple CE, Albarca Aguilera M, Meyer R, et al. VarSome: the human genomic variant search engine. Bioinformatics. 2019;35(11):1978–80. doi: 10.1093/bioinformatics/bty897 30376034 PMC6546127

[15] Li H. Minimap2: pairwise alignment for nucleotide sequences. Bioinformatics. 2018;34(18):3094–100. doi: 10.1093/bioinformatics/bty191 29750242 PMC6137996

[16] Didion JP, Martin M, Collins FS. Atropos: specific, sensitive, and speedy trimming of sequencing reads. PeerJ. 2017;5:e3720. doi: 10.7717/peerj.3720 28875074 PMC5581536

[17] Patro R, Duggal G, Love MI, Irizarry RA, Kingsford C. Salmon provides fast and bias-aware quantification of transcript expression. Nat Methods. 2017;14(4):417–9. doi: 10.1038/nmeth.4197 28263959 PMC5600148

[18] Love MI, Huber W, Anders S. Moderated estimation of fold change and dispersion for RNA-seq data with DESeq2. Genome Biol. 2014;15(12):550. doi: 10.1186/s13059-014-0550-8 25516281 PMC4302049

[19] Otto EA, Tory K, Attanasio M, Zhou W, Chaki M, Paruchuri Y, et al. Hypomorphic mutations in meckelin (MKS3/TMEM67) cause nephronophthisis with liver fibrosis (NPHP11). J Med Genet. 2009;46(10):663–70. doi: 10.1136/jmg.2009.066613 19508969

[20] Ellingwood SS, Cheng A. Biochemical and clinical aspects of glycogen storage diseases. J Endocrinol. 2018;238(3):R131–R41. doi: 10.1530/JOE-18-0120 29875163 PMC6050127

[21] Steinbacher T, Kummer D, Ebnet K. Junctional adhesion molecule-A: functional diversity through molecular promiscuity. Cell Mol Life Sci. 2018;75(8):1393–409. doi: 10.1007/s00018-017-2729-0 29238845 PMC11105642

[22] Ju M, Bai X, Zhang T, Lin Y, Yang L, Zhou H, et al. Mutation spectrum of COL1A1/COL1A2 screening by high-resolution melting analysis of Chinese patients with osteogenesis imperfecta. J Bone Miner Metab. 2020;38(2):188–97. doi: 10.1007/s00774-019-01039-3 31414283

[23] Karaca E, Harel T, Pehlivan D, Jhangiani SN, Gambin T, Coban Akdemir Z, et al. Genes that affect brain structure and function identified by rare variant analyses of mendelian neurologic disease. Neuron. 2015;88(3):499–513. doi: 10.1016/j.neuron.2015.09.048 26539891 PMC4824012

[24] Diamond T, Nema N, Wen J. Hepatic ciliopathy syndromes. Clin Liver Dis (Hoboken). 2021;18(4):193–7. doi: 10.1002/cld.1114 34745577 PMC8549716

[25] Halbritter J, Bizet AA, Schmidts M, Porath JD, Braun DA, Gee HY, et al. Defects in the IFT-B component IFT172 cause Jeune and Mainzer-Saldino syndromes in humans. Am J Hum Genet. 2013;93(5):915–25. doi: 10.1016/j.ajhg.2013.09.012 24140113 PMC3824130

[26] Bujakowska KM, Zhang Q, Siemiatkowska AM, Liu Q, Place E, Falk MJ, et al. Mutations in IFT172 cause isolated retinal degeneration and Bardet-Biedl syndrome. Hum Mol Genet. 2015;24(1):230–42. doi: 10.1093/hmg/ddu441 25168386 PMC4326328

[27] Schaefer E, Stoetzel C, Scheidecker S, Geoffroy V, Prasad MK, Redin C, et al. Identification of a novel mutation confirms the implication of IFT172 (BBS20) in Bardet-Biedl syndrome. J Hum Genet. 2016;61(5):447–50. doi: 10.1038/jhg.2015.162 26763875

[28] Yamada M, Uehara T, Suzuki H, Takenouchi T, Fukushima H, Morisada N, et al. IFT172 as the 19th gene causative of oral-facial-digital syndrome. Am J Med Genet A. 2019;179(12):2510–3. doi: 10.1002/ajmg.a.61373 31587445

[29] Whitley CB, Schwarzenberg SJ, Burke BA, Freese DK, Gorlin RJ. Direct hyperbilirubinemia and hepatic fibrosis: a new presentation of Jeune syndrome (asphyxiating thoracic dystrophy). Am J Med Genet Suppl. 1987;3:211–20. doi: 10.1002/ajmg.1320280525 3130856

[30] Schmidts M. Clinical genetics and pathobiology of ciliary chondrodysplasias. J Pediatr Genet. 2014;3(2):46–94. doi: 10.3233/PGE-14089 25506500 PMC4262788

[31] Lehmann P, Hohberger B, Lammer R, Mardin C. Extended ganglion cell layer thickness deviation maps with OCT in glaucoma diagnosis. Front Med (Lausanne). 2021;8:684676. doi: 10.3389/fmed.2021.684676 34150817 PMC8212507

[32] Rai RP, Liu Y, Iyer SS, Liu S, Gupta B, Desai C, et al. Blocking integrin alpha4beta7-mediated CD4 T cell recruitment to the intestine and liver protects mice from western diet-induced non-alcoholic steatohepatitis. J Hepatol. 2020;73(5):1013–22. doi: 10.1016/j.jhep.2020.05.047 32540177 PMC7839272

[33] Gupta B, Liu Y, Chopyk DM, Rai RP, Desai C, Kumar P, et al. Western diet-induced increase in colonic bile acids compromises epithelial barrier in nonalcoholic steatohepatitis. FASEB J. 2020;34(5):7089–102. doi: 10.1096/fj.201902687R 32275114 PMC7831197

[34] Rahman K, Desai C, Iyer SS, Thorn NE, Kumar P, Liu Y, et al. Loss of junctional adhesion molecule A promotes severe steatohepatitis in mice on a diet high in saturated fat, fructose, and cholesterol. Gastroenterology. 2016;151(4):733–46 e12. doi: 10.1053/j.gastro.2016.06.022 27342212 PMC5037035

[35] Sambrotta M, Strautnieks S, Papouli E, Rushton P, Clark BE, Parry DA, et al. Mutations in TJP2 cause progressive cholestatic liver disease. Nat Genet. 2014;46(4):326–8. Epub 2014/03/13. doi: ng.2918 [pii] 10.1038/ng.2918. doi: 10.1038/ng.2918 24614073 PMC4061468

[36] Hadj-Rabia S, Baala L, Vabres P, Hamel-Teillac D, Jacquemin E, Fabre M, et al. Claudin-1 gene mutations in neonatal sclerosing cholangitis associated with ichthyosis: a tight junction disease. Gastroenterology. 2004;127(5):1386–90. doi: 10.1053/j.gastro.2004.07.022 15521008

