# Correction: Exome sequencing reveals *IFT172* variants in patients with non-syndromic cholestatic liver disease

**DOI:** 10.1371/journal.pone.0318705

**Published:** 2025-01-30

**Authors:** Magdaléna Neřoldová, Elżbieta Ciara, Janka Slatinská, Soňa Fraňková, Petra Lišková, Radana Kotalová, Janka Globinovská, Markéta Šafaříková, Lucie Pfeiferová, Hana Zůnová, Lenka Mrázová, Viktor Stránecký, Alena Vrbacká, Ondřej Fabián, Eva Sticová, Daniela Skanderová, Jan Šperl, Marta Kalousová, Tomáš Zima, Milan Macek, Joanna Pawlowska, A. S. Knisely, Stanislav Kmoch, Milan Jirsa

There are errors in the S3 Table. In the S3B Table, the predicted translation of the heterozygous ABCB11 variant c.3589C>G found in the patient F39CE53 should shave been p.(Leu1197Val). In S3C Table, the variant found in the patient M11RO526 should be c.1798A>T and in patient F4RO528 should be c. 2545T>C. Please view the correct S3 Table below.

## Supporting information

S3 Table Variants detected by initial Sanger sequencing in 60 excluded patients. (DOCX)
