# Supplementary material for: Exome sequencing reveals IFT172 variants in patients with non-syndromic cholestatic liver disease
Source: PLoS One. 2023 Jul 20;18(7):e0288907. doi: 10.1371/journal.pone.0288907 (PMC10358992; doi:10.1371/journal.pone.0288907)
Supplement: S1 File — (DOCX) [file pone.0288907.s001.docx]

**List of referring centres**

*Australia:* Princess Margaret Hospital, Perth, Royal Children's Hospital, Brisbane, Sydney Children´s Hospital, Sydney

*Czech Republic:* Centrum Kardiovaskulární a Transplantační Chirurgie, Brno, Fakultní Nemocnice Brno, Fakultní Nemocnice Královské Vinohrady, Fakultní Nemocnice Motol, Fakultní Nemocnice Na Bulovce, Fakultní Nemocnice Olomouc, Fakultní nemocnice Ostrava, Fakultní Nemocnice Plzeň, Chrudimská Nemocnice, Institut Klinické a Experimentální Medicíny, Praha, Masarykova Nemocnice v Ústí nad Labem, Nemocnice Benešov, Nemocnice České Budějovice, Nemocnice Mělník, Nemocnice Pardubice, Ústřední Vojenská Nemocnice, Praha, Všeobecná Fakultní Nemocnice, Praha

*Germany:* Internistische Gemeinschaftspraxis für Verdauungs- und Stoffwechselerkrankungen, Leipzig, Medizinische Klinik II, Osnabrück

*Greece:* Aghia Sophia Children´s Hospital, Athens, Aristotle University Medical School, Thessaloniki, University General Hospital of Patras

*Hungary:* Gyermekklinika Budapest

*Italy:* Universita degli Studi di Roma - Sapienza, Roma

*New Zealand:* Starship Hospital, Auckland

*Poland;* Centrum Zdrowia Dziecka, Marii Sklodowskiej-Curie, Warszawa, Narodowy Instytut Onkologii im. Marii Sklodowskej-Curie, Warszawa

*Slovakia:* Fakultná Nemocnica F.D.Roosevelta Banská Bystrica, Fakultná Nemocnica s Poliklinikou Bratislava, Nemocnica Poprad, Univerzitná Nemocnica L. Pasteura, Košice

*Thailand:* Bangkok Hospital Medical Center, Bangkok

*United Arab Emirates:* Tawam Hospital, Al Ain

*United Kingdom:* Imperial College, London, King's College Hospital, London
