# Supplementary material for: Exome sequencing reveals IFT172 variants in patients with non-syndromic cholestatic liver disease
Source: PLoS One. 2023 Jul 20;18(7):e0288907. doi: 10.1371/journal.pone.0288907 (PMC10358992; doi:10.1371/journal.pone.0288907)
Supplement: S1 Table — (DOCX) [file pone.0288907.s002.docx]

**S1 Table. Antibodies**

| **Name** | **Supplier** | **Cat no.** | **Clone no.** |
| --- | --- | --- | --- |
| anti-cytokeratin 7 | Agilent Technologies Denmark ApS, Glostrup, Denmark | M7018 | OV-TL 12/30 |
| anti-ABCB4 | Kamiya Biomed. Co. Seattle, WA | MC-224 | P_3_II-26 |
| anti-ABCB11 | Merck KGaA, Darmstadt, Germany | HPA019035 | polyclonal |
| anti-alpha-1-antitrypsin | Agilent Technologies Denmark ApS, Glostrup, Denmark | A0012 | polyclonal |
