# Supplementary material for: Exome sequencing reveals IFT172 variants in patients with non-syndromic cholestatic liver disease
Source: PLoS One. 2023 Jul 20;18(7):e0288907. doi: 10.1371/journal.pone.0288907 (PMC10358992; doi:10.1371/journal.pone.0288907)
Supplement: S2 Table — (DOCX) [file pone.0288907.s003.docx]

**S2 Table.** **Sequence based reagents**

| **Name** | **Forward (5’ – 3’)** | **Reverse (5’ – 3’)** |
| --- | --- | --- |
| *JAG1* ex1 | agtcgtgcatgctccaatc | acgagtgcggaagaaatcc |
| *JAG1* ex2 | agggagtcgccacctctatac | caagccaaagccctttaaatc |
| *JAG1* ex3 | gagtcatttgtcaacctgggaag | cctcttgccttctgttctccc |
| *JAG1* ex4 | aagccaaaatggtcagggaag | atcccaccccacctgagatag |
| *JAG1* ex5 | aaaatcgtggaatttgcagac | aaagctttcttgttctccaagg |
| *JAG1* ex6 | agcctggactttgcagtcttc | acccacacagcattcaaggag |
| *JAG1* ex7 | ggtgggatggcactaatttgg | tcgctgagctgtctctaaccac |
| *JAG1* ex8 | gtgtgggattcggttggag | accgagacattcacactggac |
| *JAG1* ex9 | ggtgtctggctcttcaatgac | ctcgtcttctgtaatggctttg |
| *JAG1* ex10 | cagacaaactctggcctgttc | agcaagtcggctacccaag |
| *JAG1* ex11 | atctcacggaaagcacacagg | cacgaggctggggtaacataag |
| *JAG1* ex12 | aaccagctaaaccgcaacag | ttccagacacaagagctgagg |
| *JAG1* ex13 | ccagagaagttatcgtgacacc | aacaaggggcagtggtagtaag |
| *JAG1* ex14 | cttaggaatgccgcatctg | actctagcctgggcaacaac |
| *JAG1* ex15 | caggtgcattgtgtcaggag | aaatcactgcggtcttgcttc |
| *JAG1* ex16 | tcatttccatgggacttccag | cgtggcctcatcacactgac |
| *JAG1* ex17 | tggtagttggccttggatgc | cgaccaccctccctgagtatc |
| *JAG1* ex18 | tgcccctccagactgtttc | aagtccccaagggtgtcag |
| *JAG1* ex19 | cttagggctaagaccgctttc | gtgtgtgtgagaatcacccag |
| *JAG1* ex20 | tggtgacatgtgagtgattgg | gatgaaagtcttggggtgagg |
| *JAG1* ex21 | caatctcaaaacattgccacac | gtcaaatggtgactgcaaagc |
| *JAG1* ex22_23 | cagcaaaggcaggaagtacac | agatcctagctcatggcattc |
| *JAG1* ex24_25 | tgaacttagccagcctcaaag | actgccagataatccctcgac |
| *ABCB11* ex1 | ccaaattgttctttcgtttgg | tgctccttgaaacttgaccag |
| *ABCB11* ex2 | aatttcttaatgactgcgttgc | tgaagtgcaatgtgcatgaaag |
| *ABCB11* ex3 | agccagtggggattttctttc | ggataggatttgtatgctttatgagc |
| *ABCB11* ex4 | cccatgaaatttggtgttgag | tccagctcagccagtaaaatc |
| *ABCB11* ex5 | aatccctctatatccacaaatg | tggcaacacattgcatctc |
| *ABCB11* ex6 | ttactttcccccttttctcaac | aagccatgccacatatgaaag |
| *ABCB11* ex7 | tgctatccaagggtgataggg | caggaaaagggactcaagcttc |
| *ABCB11* ex8 | ccatgccacatgttaaatgaatc | ccgctttgcacaaactgag |
| *ABCB11* ex9 | ctgaagctgctctgtgtttgc | cctgaaggcaccaaagtaataaac |
| *ABCB11* ex10 | tctctgcgttaacatggaagac | ggtaaattcttcaggagttcattctg |
| *ABCB11* ex11 | acattgcagagatacgccaaag | aatgagcaatttgtggttatgc |
| *ABCB11* ex12 | gatttcagtggacgttgctttg | ctatgcatgccaggacagtctc |
| *ABCB11* ex13 | ttctgcccattggtcaagtatg | tcaggcatgaaactaaaacatgg |
| *ABCB11* ex14 | aagtcggagcagtgaaaattcg | tttcctgcagcagcacaag |
| *ABCB11* ex15 | tggattctgaaatgatgcaaag | ataacgcctgccagagttgttg |
| *ABCB11* ex16 | cagaatgttttggcatttgacatag | cagagtttccttgttgtacctgag |
| *ABCB11* ex17 | acttggacaccagttgatcctg | gcttaaagggtacccaacagtc |
| *ABCB11* ex18 | ctgtgaatgccaaaggatctg | tgtgtgatggaggcttagg |
| *ABCB11* ex19_20 | gatccacagcttacattaggg | aaacatgcaggtgattgtcag |
| *ABCB11* ex21 | ggtctgacctttttgtgtaatgg | gacagcttccttcagtctcttcg |
| *ABCB11* ex22 | ctgccatttgcaccatctaatc | tggtttgctaagcagcaaaaag |
| *ABCB11* ex23 | tcaaggatatttggtcctttcc | ccacaccatcccctgaca |
| *ABCB11* ex24 | ctttggcagcatggtttgaag | aacttgaagcccacttttaggg |
| *ABCB11* ex25 | accaaatgtcctgcataacacc | gctcaacctgtacactctggtc |
| *ABCB11* ex26 | acagaggagaccttgacatgag | tccacaaagtattgccaatttc |
| *ABCB11* ex27 | ttgggtttgccgtcaagtatag | actgagtggtggctgagctg |
| *ATP8B1* ex1 | gcagcatgcaggcagtattc | atcatctttggcacgtgtgg |
| *ATP8B1* ex2 | caacatttgaatctggggaaga | acgtggaaggcaggtgtagc |
| *ATP8B1* ex3 | gctgtgggacttgtgacttttg | tgctccctttccctgttcata |
| *ATP8B1* ex4_5 | gatgacggtgatgagacttgg | ttatctctgaatgtgtttctaggc |
| *ATP8B1* ex6_7 | tgtgacttgcatattttccagaatc | acaatgccccgatttcagtg |
| *ATP8B1* ex8_9 | ttcatgtccaggtatggctaatg | ggacagaaaagcaatcccctcta |
| *ATP8B1* ex10 | cccaactcagcctccaaaac | ttcctgcatttgaagcttgg |
| *ATP8B1* ex11 | tttgggaacaaaaccttaccttc | tgttgggagaaggtacaaca |
| *ATP8B1* ex12 | aagcagcttgctctcgtctg | caatgccaggagacaggcta |
| *ATP8B1* ex13 | ttagcctgtccaggccgtat | aggagcagggaagaggcaac |
| *ATP8B1* ex14 | ccttgcctttgaagaaaagttca | tgcatttgagccataagcag |
| *ATP8B1* ex15 | gctttgcagcccatctgac | ctggctgctttatcctgatgc |
| *ATP8B1* ex16 | ggctacagtgagccgatactga | ctctttccaccccaactattgc |
| *ATP8B1* ex17 | cctggatgataaagccagacct | cttcttccattgtgccagtgtc |
| *ATP8B1* ex18 | agcccttctttttacaggttgg | tcatcttgggcaaaggaaac |
| *ATP8B1* ex19 | ggcagtgggagtgagatgct | gccccaagtgacacatcgta |
| *ATP8B1* ex20 | gcgtgatagcgtgtgcctat | ccaagataaccacttttacctcttt |
| *ATP8B1* ex21 | aaatcttgggaatggtactcc | atccaccccctacacattcc |
| *ATP8B1* ex22 | gagggatggtgagcaagagc | gccccaactgtaaggagacac |
| *ATP8B1* ex23 | aagcagatgagtatgtgctaactagg | accagaagaatgcattgaaacg |
| *ATP8B1* ex24_25 | cagcaggctgcaactttttgt | ggtaccaatagacactgaatacgg |
| *ATP8B1* ex26 | ccaccacacctggcgaaata | tgcaggaaacgtgctgttg |
| *ATP8B1* ex27 | tcatttctgtcaaccgtctgg | tgatgaatgcaattcacacacac |
| *ABCB4* ex1 | ccctggctgcaacggtagg | ctgctgattggcgtgtaacg |
| *ABCB4* ex2 | accttcgacagttacttttgtgc | tcaatacctcaaatttgaataaaagg |
| *ABCB4* ex3 | caaagaatatataattatggaagagg | ggtaatgaatagcaaaatcaactcc |
| *ABCB4* ex4 | agatttttaaaaacctggcaatg | tgggtaaagagtacacgttatttg |
| *ABCB4* ex5 | aaagaaaagagaaggtatttaatagagc | tttccttgacatattttcacacag |
| *ABCB4* ex6 | actgaaacctcctgcctgtaacc | agacctgaacaggtacaagtacg |
| *ABCB4* ex7 | cagagtgcctttaaacttttctcc | atgcgagaagggttaatattagg |
| *ABCB4* ex8 | cgagtgtgactcggactatgg | actggacagtggaaagattcacc |
| *ABCB4* ex9 | aaaaggaaaggataaacctaaacttaat | tgattcaaaaatatgcaaactaaagc |
| *ABCB4* ex10 | acattccaggtcctatttttgg | aaagtatcaaataagggaatataacc |
| *ABCB4* ex11 | atcgaattattcccattttaggg | cgcaaatttgttactgaaaaacc |
| *ABCB4* ex12 | tgaaccaagttaaatattaggtagg | aaacctttgaagaataaactcagtcc |
| *ABCB4* ex13 | ttttaaatttaagttacttcaagagc | cactggcaagaatcttcaatagg |
| *ABCB4* ex14 | gacagtgtgccaatactgtaacc | ttgctcagtatagcattcactgg |
| *ABCB4* ex15 | acactttaatgtcttgatattctttcag | caaagagtatggctcatagtagc |
| *ABCB4* ex16 | ctggtaactgttgtgtcatcacc | gcttttctcaaaagggatttaagg |
| *ABCB4* ex17 | ttttaaacatgtgacactcaagc | aagaatttggaagctccattagg |
| *ABCB4* ex18 | ccgtggcaactgtaaaacaac | ggggaaaacatgcatatcg |
| *ABCB4* ex19 | aggccagggactctacactgc | catcttaacaagtgtgggtatgc |
| *ABCB4* ex20 | cattctgaccaagaggctaagg | catgtctaaaaacaacacttaacacc |
| *ABCB4* ex21 | ccttcaggatacttttgacagagc | tcagccttaggaaagcactagg |
| *ABCB4* ex22 | aggtggaacttaaacccactcg | gacctcatctttggacacagg |
| *ABCB4* ex23 | ggagaaaggggatgattaagg | tggtgtaatcatcacaaacttatcc |
| *ABCB4* ex24 | ttaactggcaccagaactatacc | ggttgggccaattaaaatatagc |
| *ABCB4* ex25 | ggcctcaatggtataagtcttgg | agattttgttggcataactttgg |
| *ABCB4* ex26 | aacactctgttaagttgaaacaacg | tcatggttgacagcaaaatcc |
| *ABCB4* ex27 | tttatacaatttttgggataagg | ttgatctagaatgagacagacatacc |
| *TNFRSF1A* ex4 | tgtgttttggtgggacactg | tacaggagggggaaggaaag |
| *TMEM67* ex18 | tcaagtaatttttattataggcacaga | ttagcaggccttttaaatttgg |
| *PKHD1* ex56 | ttagcaggccttttaaatttgg | cctttgtgcttggagaagct |
| *PKHD1* ex60 | attccacccactctggttc | aggcctacgttgaccaactc |
| *PPOX* ex4 | agctggggaggtatgtcagg | agctctgcagctggtttagg |
| *TP63* ex6 | ttcttttgccaccaacatcc | cactgtcttagttcttgtcatcaaa |
| *IFT172* ex2 | tggcattctagaccagcac | catcagcataggaaatggca |
| *F11R* ex2 | agagccagagggagcataag | cttccttcggctcattttga |
| *COL1A2* ex16 | tgtcatgccactgtaagcaac | tgtccattttgaagggaagg |
| *COL2A1* ex6 | tctctttctccgtcccttcc | gtttaagggcctggtcacct |
| *ZNF423* ex4 | gtcaatgggctgccctac | ggaaaggcacccagacat |
| *IFT172* ex2_20 | tggcattctagaccagcac | ggaagaggggatatgaggga |
| *F11R* cDNA ex1_10 | gtctgttcccaggagtccttc | agacaaataaggcatcctgtg |
| *F11R* cDNA ex1_3 | gtctgttcccaggagtccttc | taggcttggatggaggcaca |
