# Supplementary material for: Exome sequencing reveals IFT172 variants in patients with non-syndromic cholestatic liver disease
Source: PLoS One. 2023 Jul 20;18(7):e0288907. doi: 10.1371/journal.pone.0288907 (PMC10358992; doi:10.1371/journal.pone.0288907)
Supplement: S4 Table — (DOCX) [file pone.0288907.s005.docx]

**S4A Table. Clinical diagnosis of 34 enrolled patients sequenced initially by Sanger method**

| **Clin. dx.** | **Patient counts** | |
| --- | --- | --- |
|  | **Gene / Heterozygotes** | **Negative** |
| Aalgille syndrome | *JAG1* / 0 | 3 |
| low-GGT PFIC | *ABCB11* / 2 | 11 |
| low-GGT BRIC | *ABCB11* / 1 | 4 |
| low-GGT ICP | *ABCB11* / 1 | 2 |
| low-GGT CIC | *ABCB11* / 1 | 0 |
| high-GGT PFIC | *ABCB4* / 3 | 6 |
| Total | 8 | 26 |

PFIC – progressive familial intrahepatic cholestasis, BRIC – benign recurrent intrahepatic cholestasis, ICP – intrahepatic cholestasis of pregnancy, CIC – contraceptives-associated intrahepatic cholestasis.

**S4B Table. Variants detected in *ABCB11* and *ATP8B1* by Sanger sequencing in 5 enrolled heterozygotes with low-GGT intrahepatic cholestasis.**

| **Patient ID** | **Clin. dx.** | **Variants in *ABCB11* and *ATP8B1*** | **HGMD** | **ACMG** | **Origin** |
| --- | --- | --- | --- | --- | --- |
| M2ME312 | PFIC | *ABCB11* c.1268A>G p.(His423Arg) rs147522210 | NA | VUS | pat |
| F0CE558 | PFIC | *ABCB11* c.1445A>G p.(Asp482Gly) rs72549402 | CM980247 | P | mat |
| M17CE365 | BRIC | *ABCB11* c.1924G>T p.(**Glu642***) | NA | LP | mat |
| F23CE336 | CIC | *ABCB11* c.74C>A p.(**Ser25***) | CM067622 | LP | pat |
| F28CE533 | ICP | *ABCB11* c.908G>A p.(Arg303Lys) | CM081486 | VUS | pat |

Patients are sorted according to their clinical diagnosis. Origin of the variants was assessed by targeted re-sequencing of parental genomic DNA samples when available. Nonsense or variants are **boldfaced**. ACMG classification criteria were valid in week 20, 2023. Patient ID includes gender identifier (M – male, F – female), patient age in years at specimen receipt, origin (CE – Central Europe, ME – Middle East), and DNA sample number. Clin. dx. – clinical diagnosis, HGMD – Human Gene Mutation Database accession number, PFIC – progressive familial intrahepatic cholestasis, BRIC – benign recurrent intrahepatic cholestasis, ICP – intrahepatic cholestasis of pregnancy, CIC – contraceptives-associated intrahepatic cholestasis, rs – dbSNP accession number, NA – not available.

**S4C Table. Variants detected in *ABCB4* by Sanger sequencing in 3 enrolled heterozygotes with suspected genetic high-GGT intrahepatic cholestasis.**

| **Patient ID** | **Clin. dx.** | **Variants in *ABCB4*** | **HGMD** | **ACMG** | **Origin** |
| --- | --- | --- | --- | --- | --- |
| F15CE43 | PFIC | c.101C>T p.(Thr34Met) rs142794414 | CM122249 | LB | NA |
| F3PA342 | PFIC | c.139C>T p.(**Arg47***) rs376926391 | CM082466 | P | pat |
| F42CE158 | PFIC | c.1501G>T p.(**Glu501***) rs1562976167 | CM147043 | P | NA |

Origin of the variants was assessed by targeted re-sequencing of parental genomic DNA samples when available. Nonsense variants are **boldfaced**. ACMG classification criteria were valid in week 20, 2023. Patient ID includes gender identifier (M – male, F – female), patient age in years at specimen receipt, origin (CE – Central Europe, PA – Australian Pacific), and DNA sample number. Clin. dx. – clinical diagnosis, HGMD – Human Gene Mutation Database accession number, rs – dbSNP accession number, NA – not available.
