# Supplementary material for: Exome sequencing reveals IFT172 variants in patients with non-syndromic cholestatic liver disease
Source: PLoS One. 2023 Jul 20;18(7):e0288907. doi: 10.1371/journal.pone.0288907 (PMC10358992; doi:10.1371/journal.pone.0288907)
Supplement: S5 Table — (DOCX) [file pone.0288907.s006.docx]

**S5A Table**. **Expression of 66 genes from the Emory Genetics Laboratory (EGL) Neonatal and Adult Cholestasis Panel** **(EGL Genetics, Tucker, GA, used in Karpen S *et al*., J Pediatr Gastroenterol Nutr 2021;72:654-660)** **in nasopharyngeal swab**.

| **Gene** | **Expressed** | **Gene** | **Expressed** | **Gene** | **Expressed** | **Gene** | **Expressed** |
| --- | --- | --- | --- | --- | --- | --- | --- |
| *ABCB11* | Yes | *DGUOK* | Yes | *NPHP3* | Yes | *PKHD1* | no |
| *ABCB4* | no | *DHCR7* | Yes | *NPHP4* | Yes | *POLG* | Yes |
| *ABCC2* | no | *EHHADH* | Yes | *NR1H4* | no | *SCP2* | Yes |
| *ABCG5* | no | *FAH* | Yes | *PENO1* | Yes | *SERPINA1* | Yes |
| *ABCG8* | no | *GPBAR1* | no | *PENO10* | Yes | *SLC10A1* | no |
| *AKR1D1* | no | *HNF1B* | no | *PENO11B* | Yes | *SLC10A2* | no |
| *ALDOB* | no | *HSD17B4* | Yes | *PENO12* | Yes | *SLC25A13* | Yes |
| *AMACR* | Yes | *HSD3B7* | Yes | *PENO13* | Yes | *SLC27A5* | no |
| *ATP8B1* | Yes | *INVS* | Yes | *PENO14* | Yes | *SMPD1* | Yes |
| *BAAT* | no | *JAG1* | Yes | *PENO16* | Yes | *TJP2* | Yes |
| *CC2D2A* | Yes | *LIPA* | Yes | *PENO19* | Yes | *TMEM216* | Yes |
| *CFTR* | Yes | *MKS1* | Yes | *PENO2* | Yes | *TRMU* | Yes |
| *CLDN1* | Yes | *MPV17* | Yes | *PENO26* | Yes | *UGT1A1* | no |
| *CYP27A1* | Yes | *NOTCH2* | Yes | *PENO3* | Yes | *VIPAS39* | Yes |
| *CYP7A1* | no | *NPC1* | Yes | *PENO5* | Yes | *VPS33B* | Yes |
| *CYP7B1* | no | *NPC2* | Yes | *PENO6* | Yes |  |  |
| *DCDC2* | Yes | *NPHP1* | Yes | *PENO7* | Yes |  |  |

Yes - mRNA expressed, no - mRNA absent

**S5B Table. Expression of 123 genes from the Emory Genetics Laboratory (EGL) Ciliopathy Panel (EGL Genetics, Tucker, GA)** **in nasopharyngeal swab**.

| **Gene** | **Expressed** | **Gene** | **Expressed** | **Gene** | **Expressed** | **Gene** | **Expressed** |
| --- | --- | --- | --- | --- | --- | --- | --- |
| *ACVR2B* | no | *DFNB31* | Yes | *NEK1* | Yes | *SCNN1A* | Yes |
| *AHI1* | Yes | *DNAAF1* | Yes | *NEK8* | Yes | *SCNN1B* | Yes |
| *AIPL1* | no | *DNAAF2* | Yes | *NKX2-5* | no | *SCNN1G* | Yes |
| *ARL13B* | Yes | *DNAAF3* | Yes | *NME8* | no | *SDCCAG8* | Yes |
| *ARL6* | Yes | *DNAH11* | Yes | *NODAL* | no | *SPATA7* | Yes |
| *ATXN10* | Yes | *DNAH5* | Yes | *NPHP1* | Yes | *TCTN1* | Yes |
| *B9D1* | Yes | *DNAI1* | Yes | *NPHP3* | Yes | *TCTN2* | Yes |
| *B9D2* | Yes | *DNAI2* | Yes | *NPHP4* | Yes | *TMEM138* | Yes |
| *BBS1* | Yes | *DNAL1* | Yes | *OFD1* | Yes | *TMEM216* | Yes |
| *BBS10* | Yes | *DYNC2H1* | Yes | *PCDH15* | no | *TMEM231* | Yes |
| *BBS12* | Yes | *EVC* | no | *PKD2* | Yes | *TMEM237* | Yes |
| *BBS2* | Yes | *EVC2* | no | *PKHD1* | no | *TMEM67* | Yes |
| *BBS4* | Yes | *FOXH1* | no | *RD3* | no | *TOPORS* | Yes |
| *BBS5* | Yes | *GDF1* | no | *RDH12* | no | *TRIM32* | Yes |
| *BBS7* | Yes | *GLIS2* | Yes | *RPE65* | no | *TSC1* | Yes |
| *BBS9* | Yes | *GPR98* | Yes | *RPGR* | Yes | *TSC2* | Yes |
| *C2orf71* | no | *GUCY2D* | no | *RPGRIP1* | no | *TTC21B* | Yes |
| *C5orf42* | Yes | *HYLS1* | Yes | *RPGRIP1L* | Yes | *TTC8* | Yes |
| *CC2D2A* | Yes | *IFT43* | Yes | *RSPH4A* | Yes | *TULP1* | no |
| *CCDC28B* | no | *IFT80* | Yes | *RSPH9* | Yes | *UMOD* | no |
| *CCDC39* | Yes | *IMPDH1* | Yes | *SCNN1A* | Yes | *USH1C* | Yes |
| *CCDC40* | Yes | *INVS* | Yes | *SCNN1B* | Yes | *USH1G* | no |
| *CDH23* | no | *IQCB1* | Yes | *SCNN1G* | Yes | *USH2A* | no |
| *CEP164* | Yes | *KCNJ13* | no | *SDCCAG8* | Yes | *VHL* | Yes |
| *CEP290* | Yes | *KIF7* | no | *SPATA7* | Yes | *WDPCP* | Yes |
| *CEP41* | Yes | *LCA5* | Yes | *RPE65* | no | *WDR19* | Yes |
| *CFTR* | Yes | *LEFTY2* | no | *RPGR* | Yes | *WDR35* | Yes |
| *CLRN1* | no | *LRAT* | no | *RPGRIP1* | no | *XPNPEP3* | Yes |
| *CRB1* | no | *MKKS* | Yes | *RPGRIP1L* | Yes | *ZIC3* | no |
| *CRELD1* | Yes | *MKS1* | Yes | *RSPH4A* | Yes | *ZNF423* | no |
| *CRX* | no | *MYO7A* | no | *RSPH9* | Yes |  |  |

Yes – mRNA expressed, no – mRNA absent
