# Supplementary material for: Exome sequencing reveals IFT172 variants in patients with non-syndromic cholestatic liver disease
Source: PLoS One. 2023 Jul 20;18(7):e0288907. doi: 10.1371/journal.pone.0288907 (PMC10358992; doi:10.1371/journal.pone.0288907)
Supplement: S1 Fig — (PDF) [file pone.0288907.s007.pdf]

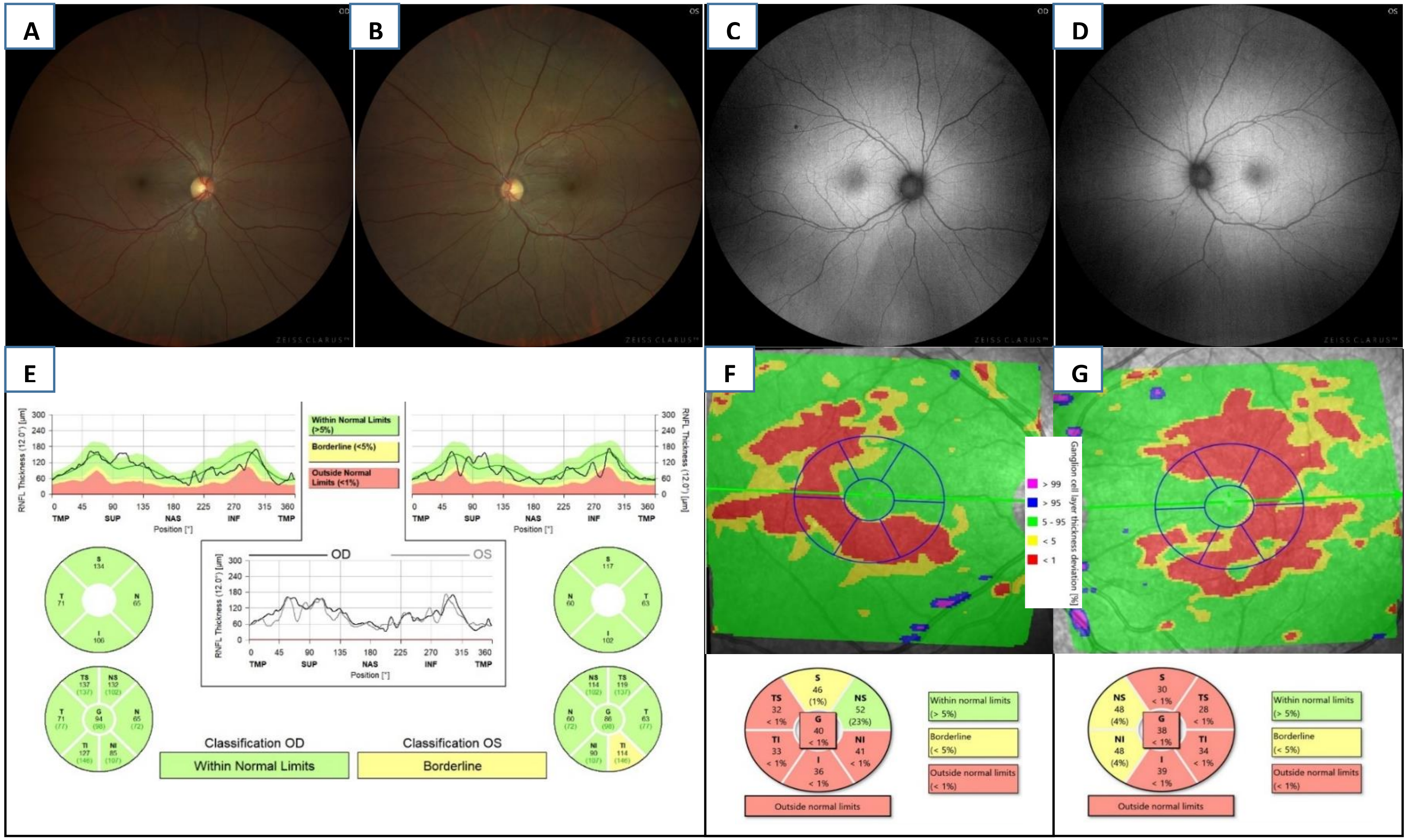

S1 Fig. Retinal findings in index case M26RO684 homozygous for the *IFT172* rs780205001 variant. Wide-field fundus photographs and fundus autofluorescence images of the right (**A**, **C**) and left (**B**, **D**) eyes document the appearance of the retina and the optic discs without visible abnormalities. Normal retinal nerve fiber layer thickness in the right eye (**E**), and borderline thickness in the left eye (**F**). Ganglion cell layer deviation thickness map documenting thinning in the macular region in the right eye (**F**), more advanced in the left eye (**G**). Red regions exhibit statistically significant thinning (< 1st percentile).
