# Supplementary material for: Exome sequencing reveals IFT172 variants in patients with non-syndromic cholestatic liver disease
Source: PLoS One. 2023 Jul 20;18(7):e0288907. doi: 10.1371/journal.pone.0288907 (PMC10358992; doi:10.1371/journal.pone.0288907)
Supplement: S2 Fig — (PDF) [file pone.0288907.s008.pdf]

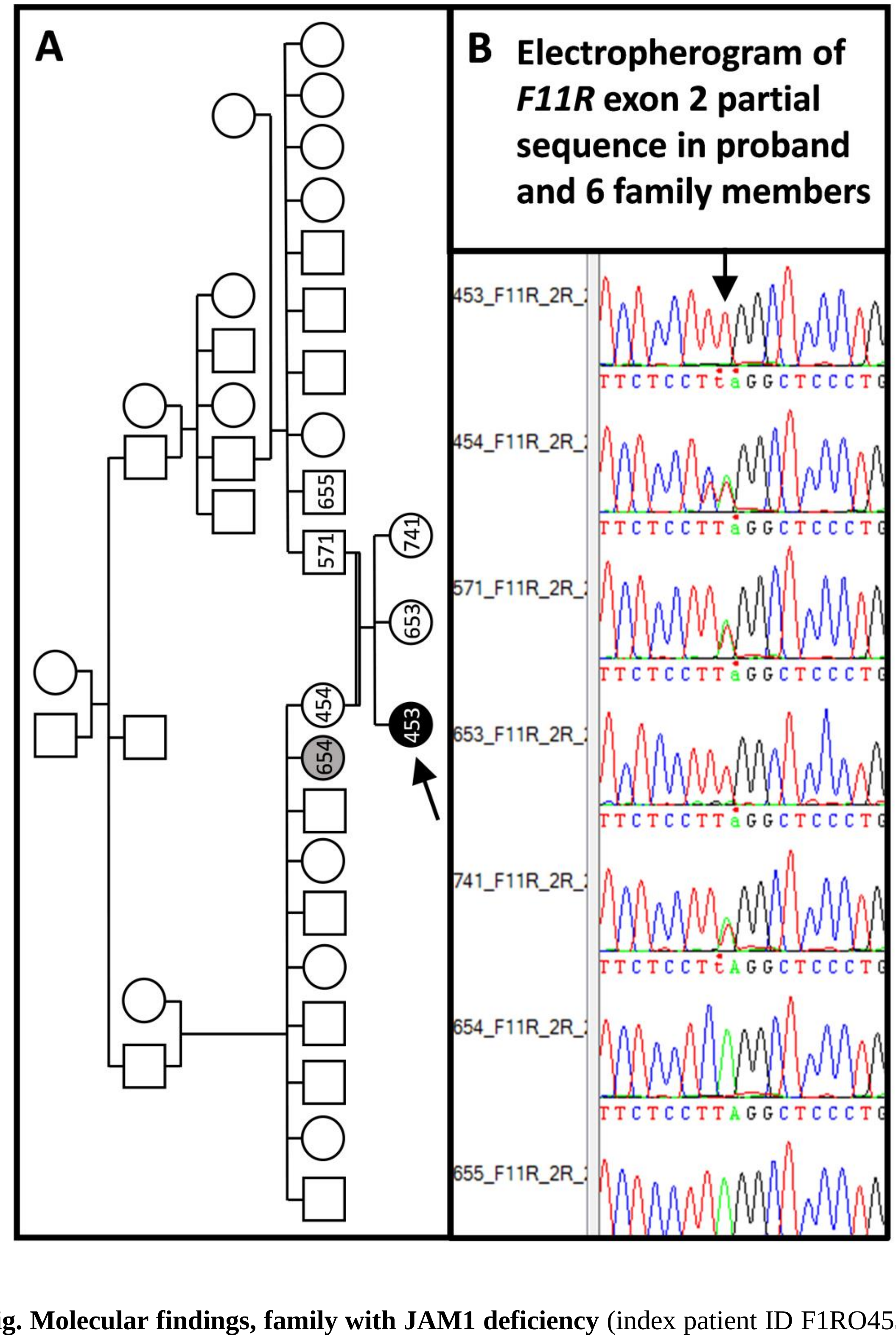

S2 Fig. Molecular findings, family with JAM1 deficiency (index patient ID F1RO453). (**A**) Pedigree; arrow, index patient. (**B**) Sanger-sequencing electropherogram of the index patient and relatives. Arrow, *F11R* c.65-2A>T splice-site variant.
