# Supplementary material for: Correction: Exome sequencing reveals IFT172 variants in patients with non-syndromic cholestatic liver disease
Source: PLoS One. 2025 Jan 30;20(1):e0318705. doi: 10.1371/journal.pone.0318705 (PMC11781684; doi:10.1371/journal.pone.0318705)
Supplement: S3 Table — (DOCX) [file pone.0318705.s001.docx]

**S3A Table. Variants detected in *JAG1* by Sanger sequencing in 19 of 22 families with clinically suspected Alagille syndrome.**

| **Patient ID** | **Variants in *JAG1*** | **HGMD** | **ACMG** | **Origin** |
| --- | --- | --- | --- | --- |
| M14CE1 | c.327_330delCAAG p.(**Lys110Profs*50**) | CD1513418 | LP | mat |
| M2CE6 | c.402G>T p.(Leu134Phe) | CM1513421 | VUS | NA |
| M0CE626 | c.551G>A p.(Arg184His) rs121918351 | CM981095 | P | mat |
| M0CE492 | c.851G>A p.(Cys284Tyr) | NA | LP | pat |
| M3CE2 | c.879_880delTG p.(**Cys293***) | CD1513419 | LP | NA |
| F8CE3 | c.960T>A p.(**Tyr320***) | CM124879 | LP | NA |
| F10CE4 | c.1313_1314delGT p.(**Cys438Serfs*10**) | CD1513420 | LP | mat |
| M0CE657 | c.1395+1G>A p.(**?**) | CS062067 | LP | mat |
| M17CE649 | c.1566T>G p.(Cys522Trp) | NA | VUS | mat |
| M13CE5 | c.1899_1900delTG p.(**Cys633***) rs1555828321 | CD993781 | P | de novo |
| M6CE7 | c.2050delG p.(**Asp684Thrfs*59**) | CD1513422 | LP | NA |
| M0CE750 | c.2122-2125delCACGT p.(**Gln708Valfs*34**) rs727504412 | CD972288 | P | NA |
| M0CE696 | c.2372+1G>A p.(**?**) | CS030120 | LP | de novo |
| M5CE729 | c.2663dupG p.(**Arg889Thrfs*63**) | NA | LP | pat |
| F11CE8 | c.2913_2914delAC p.(**Pro972Argfs*10**) | CD1513425 | LP | mat |
| F1CE433 | c.2810G>A p.(Arg937Gln) rs145895196 | CM030058 | B | NA |
| F0CE644 | c.2917-8C>A p.(**Gly973Phefs*2**) | NA | VUS | NA |
| M0CE635 | c.3048+1G>T rs876661121 p.(**?)** | CS051687 | P | mat |
| M1CE69 | c.3164dupT p.(**Arg1056Lysfs*53**) | NA | P | de novo |

All detected variants were heterozygous. Origin of the variants was assessed in 12 families by targeted re-sequencing of parental genomic DNA samples from both parents. Predicted splice site, nonsense or frame shift variants are **boldfaced**. ACMG classification criteria were valid in week 20, 2023. Patient ID includes gender identifier (M - male, F – female), patient age in years at specimen receipt, origin (CE - Central Europe), and DNA sample number. HGMD – Human Gene Mutation Database accession number, rs – dbSNP accession number, NA – not available.

**S3B Table. Variants detected in *ABCB11* and *ATP8B1* by Sanger sequencing in 32 of 94 families with suspected genetic low-GGT intrahepatic cholestasis.**

| **Patient ID** | **Clin. dx.** | **Variants in *ABCB11* and *ATP8B1*** | **HGMD** | **ACMG** | **Origin** |
| --- | --- | --- | --- | --- | --- |
| M4CE121 | PFIC | *ATP8B1* c.2097+2T>C p.(**?**) rs387906381, HET  *ATP8B1* c.2677G>C p.(Asp893His), HET | NA CM117163 | P  LP | mat pat |
| M1PA231 | PFIC | *ABCB11* c.-27-1G>A p.(**?**) rs1272189885, HET  *ABCB11* c.2343+1G>T p.(**?**) rs774411820, HET | NA  NA | LP  P | non- mat mat |
| F0EA199 | PFIC | *ABCB11* c.90_93delGAAA p.(**Lys30Asnfs*31**), HET *ABCB11* c.249_250insT p.(**Gly84Trpfs*9**), HET | CD097190 CI097187 | LP  LP | mat pat |
| F4PA253 | PFIC | *ABCB11* c.179G>A p.(**Trp60***), HOM | CM117164 | LP | both |
| F0CE414 | PFIC | *ABCB11* c.611+1G>A p.(**?**) rs769134865, HET  *ABCB11* c.1685G>A p.(Gly562Asp), HET | CS067803 CM081503 | P  LP | pat mat |
| F0CE669 | PFIC | *ABCB11* c.612-6_617delinsCAC p.(**?**), HET  *ABCB11* c.2842C>T p.(Arg948Cys) rs1559183717, HET | NA CM081493 | LP  P | non-pat pat |
| F8CE261 | PFIC | *ABCB11* c.673A>C p.(Thr225Pro), HET  *ABCB11* c.677C>T p.(Ser226Leu) rs1382100120, HET | CM117165 CM103531 | VUS  LP | mat pat |
| M19CE273 | PFIC | *ABCB11* c.731delT p.(**Ile245Leufs*17**), HET  *ABCB11* c.1445A>G p.(Asp482Gly) rs72549402, HET | CD117167 CM980247 | LP  P | non-mat  mat |
| F1CE417 | PFIC | *ABCB11* c.732delT p.(**Ile245Leufs*17**), HET  *ABCB11* c.779G>A p.(Gly260Asp), HET | CD117167 NA | LP  VUS | pat mat |
| M0CE68 | PFIC | *ABCB11* c.851T>C p.(Val284Ala) rs200739891, HET  *ABCB11* c.1468A>G p.(Asn490Asp) rs553076953, HET | CM092759 CM081493 | VUS  VUS | pat mat |
| F21CE308 | PFIC | *ABCB11* c.858T>C p.(Val284Ala), HET  *ABCB11* c.2842C>Tp.(Arg948Cys) rs1559183717, HET | CM092759 CM081493 | P  P | pat mat |
| M0CE65 | PFIC | *ABCB11* c.937C>A p.(Arg313Ser) rs375087680**,** HET  *ABCB11* c.1445A>G p.(Asp482Gly) rs72549402, HET | CM081501  CM980247 | VUS  P | mat  pat |
| M1CE488 | PFIC | *ABCB11* c.1202C>G p.(Pro401Arg), HET  *ABCB11* c.3457C>T p.(Arg1153Cys) rs72549395, HET | NA CM980251 | VUS  P | mat  pat |
| M5CE239 | PFIC | *ABCB11* c.1381A>G p.(Lys461Glu) rs1274558905, HET  *ABCB11* c.3148C>T p.(Arg1050Cys) rs72549398, HET | CM980246  CM042275 | LP  LP | pat  pat |
| M2CE215 | PFIC | *ABCB11* c.1409G>A p.(Arg470Gln) rs1463057954, HET  *ABCB11* c.1677_1679delinsAAA  p.(Met559_Ser560delinsIleAsn), HET | CM081494  CX117168 | LP  VUS | mat  pat |
| F2CE209 | PFIC | *ABCB11* c.1445A>G p.(Asp482Gly) rs72549402, HET  *ABCB11* c.1757C>T p.(Thr586Ile) rs1321213158, HET | CM980247  CM092747 | P  VUS | mat  pat |
| F0CE194 | PFIC | *ABCB11* c.1445A>G p.(Asp482Gly) rs72549402, HET  *ABCB11* c.3458G>T p.(Arg1153Leu), HET | CM980247  NA | P  LP | pat  mat |
| F3CE629 | PFIC | *ABCB11* c.1708G>A p.(Ala570Thr) rs886043807, HOM | CM024472 | P | NA |
| F55CE429 | PFIC | *ABCB11* c.2495G>A p.(Arg832His) rs376255350, HET  *ABCB11* c.2629G>A p.(Gly877Arg) rs745557569, HET | CM107193  CM103695 | P  LP | non- mat  mat |
| F2CE358 | PFIC | *ABCB11* c.3148C>T p.(Arg1050Cys) rs72549398, HET  *ABCB11* c.3904G>T p.(**Glu1302***), HET | CM042275  CM081484 | LP  LP | NA |
| M17CE111 | BRIC | *ATP8B1* c.134A>C p.(Asn45Thr) rs146599962, HET *ATP8B1* c.1805G>A p.(Arg602Gln) rs758353144, HET | CM051009 CM117172 | B  VUS | NA |
| M19CE448 | BRIC | *ATP8B1* c.191G>T p.(Trp64Leu), HOM | NA | VUS | both |
| F59CE169 | BRIC | *ATP8B1* c.238delA p.(**Met80***), HET  *ATP8B1* c.1352T>C p.(Ile451Thr), HET | NA NA | LP  VUS | NA |
| M19CE119 | BRIC | *ATP8B1* c.1982T>C p.(Ile661Thr) rs28939686, HOM | CM980761 | P | NA |
| M29CE2 | BRIC | *ATP8B1* c.1982T>C p.(Ile661Thr) rs28939686, HET *ATP8B1* c.3122_3126delTCCTAinsACATCGATGTTGATGTTAGG p.(Val1041_Leu1042delinsAspIleAspValAspVal  Arg), HET | CM980761  CX046078 | P  VUS | pat  mat |
| M29CE274 | BRIC | *ATP8B1* c.854G>T p.(Ser285Ile) rs754073606, HET *ABCB11* c.2093G>A p.(Arg698His) rs138642043, HET | NA  CM129435 | VUS  B | NA |
| M19CE109 | BRIC | *ABCB11* c.23G>C p.(Arg8Pro), HET  *ABCB11* c.3214-3C>G p.(**?**), HET | CM117171  CS118460 | VUS  VUS | non mat mat |
| M35CE24 | BRIC | *ABCB11* c.403G>A p.(Glu135Lys) rs752992432, HET  *ABCB11* c.1757C>T p.(Thr586Ile) rs1321213158, HET | CM092737  CM092747 | LP  VUS | NA |
| F39CE53 | BRIC | *ABCB11* c.2316T>A p.(**Tyr772***), HET  *ABCB11* c.3589C>G p.(Leu1197Val), HET | CM117169 NA | P  VUS | NA |
| F11CE380 | BRIC | *ABCB11* c.650T>G p.(Met217Arg) rs776085479, HET  *ABCB11* c.1724G>A p.(Arg575Gln) rs200667815, HET | CM1213495  NA | VUS  VUS | mat  pat |
| F11CE112 | BRIC | *ABCB11* c.851T>C p.(Val284Ala) rs200739891, HET  *ABCB11* c.1445A>G p.(Asp482Gly) rs72549402, HET  *ATP8B1* c.134A>C p.(Asn45Thr) rs146599962,+ HET | CM092759 CM980247  CM051009 | VUS  P  B | mat non- mat  non-  mat |
| M20RO665 | BRIC | *ABCB11* c.1028A>G p.(Tyr343Cys), HET  *ABCB11* c.1445A>G p.(Asp482Gly) rs72549402, HET | NA  CM980247 | VUS  P | NA |

Index patients with biallelic variants in either *ATP8B1* or *ABCB11* are sorted according to their clinical diagnosis. Origin of the variants was assessed by targeted re-sequencing of parental genomic DNA samples when available. Predicted splice site, nonsense or frame shift variants are **boldfaced**. ACMG classification criteria were valid in week 20, 2023. Patient ID includes gender identifier (M – male, F – female), patient age in years at specimen receipt, origin (CE – Central Europe, RO – Roma, PA – Australian Pacific, EA – East Asia), and DNA sample number. Clin. dx. – clinical diagnosis, HGMD – Human Gene Mutation Database accession number. PFIC – progressive familial intrahepatic cholestasis, BRIC – benign recurrent intrahepatic cholestasis, rs – dbSNP accession number, HET – heterozygous state, HOM – homozygous state, non-mat – mother negative, father not available, non-pat – father negative, mother not available, both – both parents are heterozygotes, NA – not available.

**S3C Table. Variants detected in *ABCB4* by Sanger sequencing in 9 of 53 families with suspected genetic high-GGT intrahepatic cholestasis.**

| **Patient ID** | **Clin. Dx.** | **Variants in *ABCB4*** | **HGMD** | **ACMG** | **Origin** |
| --- | --- | --- | --- | --- | --- |
| F5PA211 | PFIC | c.344+2_3insTT p.(**?**), HET  c.2064+3A>T p.(**?**), HET | CI121479  NA | VUS  VUS | pat  mat |
| F17CE610 | PFIC | c.475C>T p.(**Arg159***), HET  c.959C>T p.(Ser320Phe), HET | CM075939 CM013506 | P  P | NA |
| M11RO526 | PFIC | c.833+1G>T p.(**?**), HET  c.1798A>T p.(Ile600Phe), HET | NA | LP  VUS | non-mat mat |
| F37CE736 | PFIC | c.959C>T p.(Ser320Phe) rs72552778, HET  c.1436C>T p.(Pro479Leu) rs748657435, HET | CM013506  CM105793 | P  LP | NA |
| M6CE218 | PFIC | c.1285G>A p.(Gly429Arg) rs374007494, HET  c.1742G>T p.(Gly581Val), HET  c.1954A>G p.(Arg652Gly) rs2230028, HET | NA NA CM072814 | LP  VUS  B | non-mat mat mat |
| F0EA318 | PFIC | c.1326_1327delACinsGT p.(**Ile442_Gln443delinsMet***), HOM | NA | LP | both |
| M56CE114 | PFIC | c.1436C>T p.(Pro479Leu) rs748657435, HET  c.1954A>G p.(Arg652Gly) rs2230028, HET | CM105793 CM013506 | LP  B | NA |
| F4RO528 | PFIC | c.2545T>C p.(Ser849Pro), HOM | NA | VUS | both |
| M14PA222 | PFIC | c.2783+1G>T p.(**?**), HET  Chr7:**g.(86998525)_(87236463)delN237939** RCh37/hg19, HET | NA NA | LP  P | mat pat |

Origin of the variants was assessed by targeted re-sequencing of parental genomic DNA samples when available. Predicted splice site and nonsense variants and one large deletion removing the whole *ABCB4* gene are **boldfaced**. ACMG classification criteria were valid in week 20, 2023. Patient ID includes gender identifier (M – male, F – female), patient age in years at specimen receipt, origin (CE – Central Europe, RO – Roma, PA – Australian Pacific, EA – East Asia), and DNA sample number. Clin. dx. – clinical diagnosis, HGMD – Human Gene Mutation Database accession number, PFIC – progressive familial intrahepatic cholestasis, rs – dbSNP accession number, HET – heterozygous state, HOM – homozygous state, non-mat – mother negative, father not available, both – both parents are heterozygotes, NA – not available.
